# Long-term effects of western diet consumption in male and female mice

**DOI:** 10.1038/s41598-020-71592-9

**Published:** 2020-09-07

**Authors:** Yu Hasegawa, Shin-Yu Chen, Lili Sheng, Prasant Kumar Jena, Karen M. Kalanetra, David A. Mills, Yu-Jui Yvonne Wan, Carolyn M. Slupsky

**Affiliations:** 1 Department of Food Science and Technology, University of California-Davis, One Shields Avenue, Davis, CA 95616-5270 USA; 2 Department of Nutrition, University of California-Davis, Davis, CA USA; 3 Department of Medical Pathology and Laboratory Medicine, University of California-Davis, Sacramento, CA USA; 4 Present Address: Taiwan Food and Drug Administration, Nangang District, Taipei, Taiwan; 5 Present Address: Institute of Interdisciplinary Integrative Medicine Research, Shanghai University of Traditional Chinese Medicine, Shanghai, 201203 China

**Keywords:** Metabolomics, Obesity, Microbiome

## Abstract

Long-term consumption of a diet with excessive fat and sucrose (Western diet, WD) leads to an elevated risk of obesity and metabolic syndrome in both males and females. However, there are sexual dimorphisms in metabolism which are apparent when considering the prevalence of complications of metabolic syndrome, such as non-alcoholic fatty liver disease. This study aimed to elucidate the impact of a WD on the metabolome and the gut microbiota of male and female mice at 5, 10, and 15 months to capture the dynamic and comprehensive changes brought about by diet at different stages of life. Here we show that there are important considerations of age and sex that should be considered when assessing the impact of diet on the gut microbiome and health.

## Introduction

Obesity is a growing health concern worldwide. It is induced by an imbalance of energy intake and expenditure and is associated with a number of complications such as type 2 diabetes, insulin resistance, hypertension, and cardiovascular disease^1^. While diet-induced obesity is typically studied in male animals, it is known that there are sex differences in energy metabolism, fat distribution, and sex hormone balance that may impact its consequences^2,3^. Moreover, aging has been associated with metabolic decline^3^, and it is now understood that gut microbiota can impact host energy metabolism, oxidation, and hormone production, which may affect host adiposity and the risk of obesity and other metabolic disorders^4,5^.

Here, we present the impact of a diet high in saturated fat and sucrose on the metabolome and gut microbiome of male and female wild-type C57BL/6J mice at 5, 10, and 15 months of age. ^1^H nuclear magnetic resonance (NMR) metabolomics analysis was used to assess the serum and urine metabolomes, and 16S ribosomal RNA (rRNA) sequencing was performed on fecal and cecal samples to assess the gut microbiome. We found that while there were some similarities in the response to diet, male and female mice differed with respect to their serum and urine metabolomes, as well as their fecal and cecal microbiota at all ages regardless of diet. A major finding was that fecal microbiota changed, and serum ketone bodies decreased while serum lactate and pyruvate increased with age in both male and female mice. The impact of sex was reflected in the composition of cecal microbiota, as well as the urine metabolome, where in addition to several endogenous metabolite differences between males and females, several bacterial-host co-metabolites differed. Our results emphasize the importance of sex and age when making conclusions regarding diet and its effect on metabolism and gut microbiota.

## Results

### Mice on the Western diet gained weight faster than mice on the control diet

**Figure 1:**
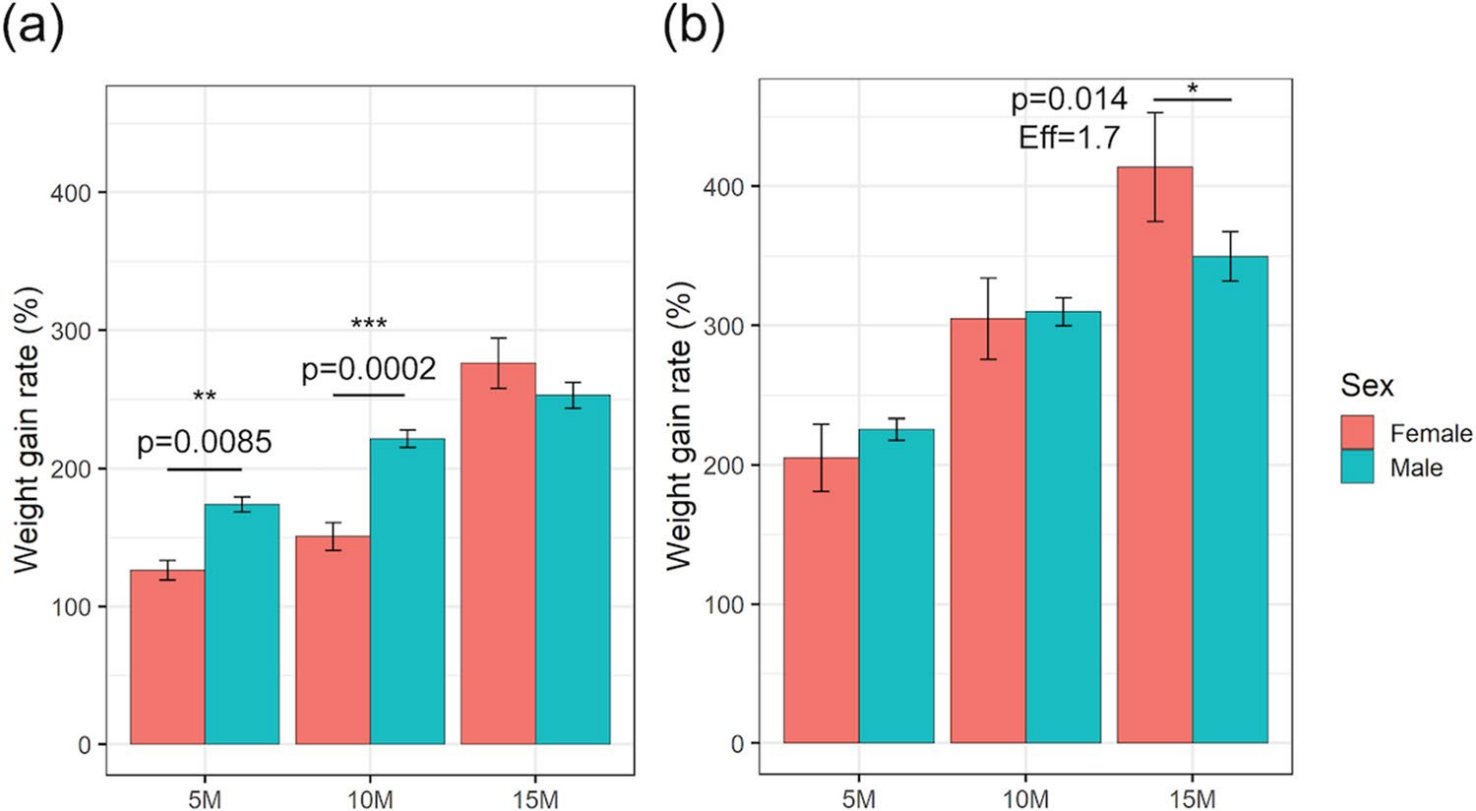
Weight gain rate (%) of mice on either (**a**) CD or (**b**) WD, for female (red) and male (blue) compared to the weight at 3 weeks old. The uncorrected* p* values from linear regression analysis as well as the effect size for female samples at 15 months of age are included. The error bar represents mean ± standard error (SE). *: *p* < 0.05; **: *p* < 0.01; ***: *p* < 0.001.

When compared with mice on a standard chow control diet (CD), both male and female mice on a Western diet (WD) showed significantly higher weight as well as rate of weight gain at each time point (*p* < 0.001 at all time points, for both sexes). On the CD, although male mice showed a significantly higher weight gain rate at 5 and 10 months of age compared to female mice (*p* < 0.01 for both time points), no statistically significant difference was found at 15 months of age (Fig. 1). On the WD, sex appeared to impact the rate of weight gain only at 15 months where female mice gained weight at a higher rate than male mice (*p* = 0.014).

### The serum metabolome was influenced primarily by age and less by diet

**Figure 2:**
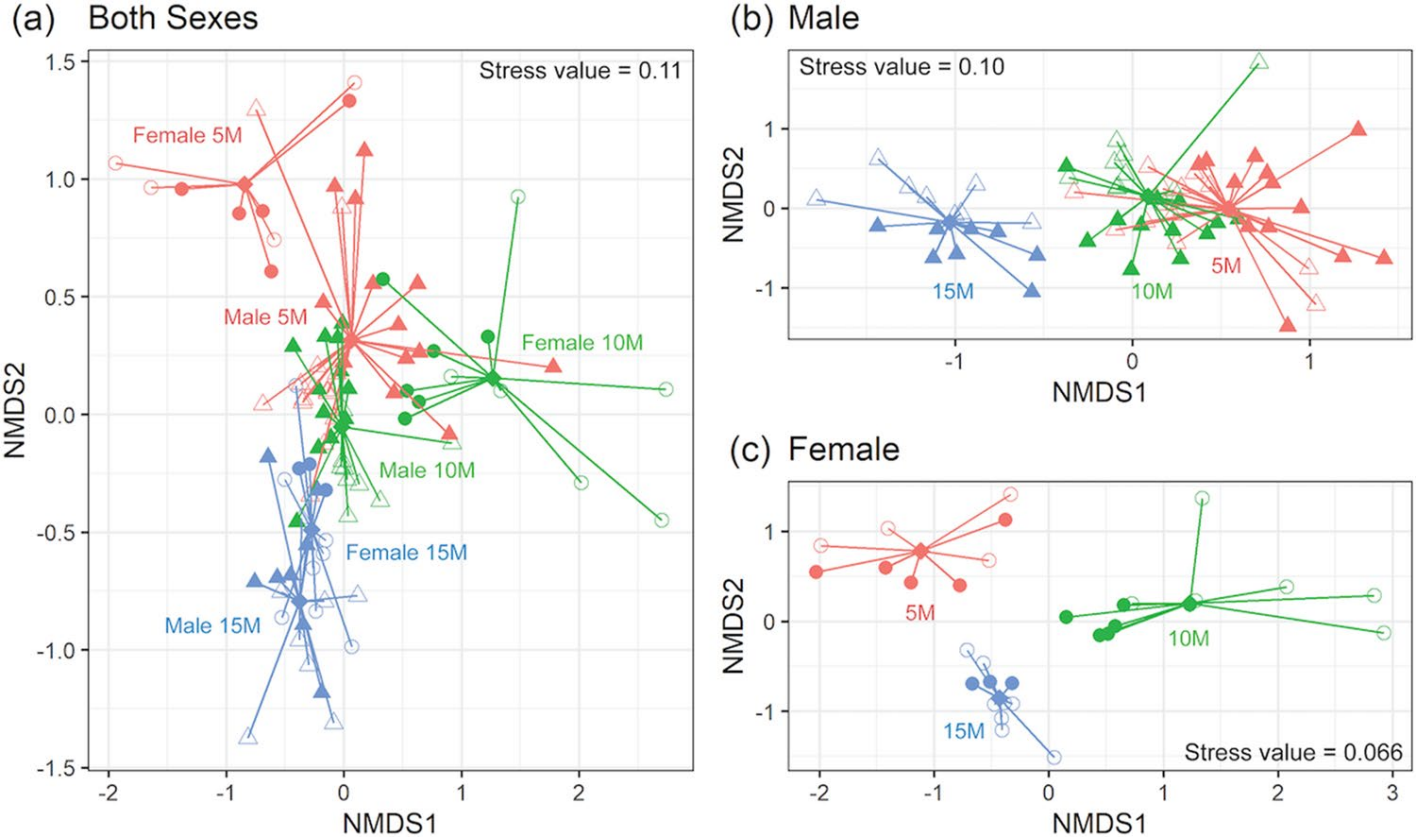
NMDS plots of the serum metabolome. The centroid of each group is noted as a diamond. (**a**) NMDS plot generated with all samples included; (**b**) NMDS plot generated with only male samples; (**c**) NMDS plot generated with only female samples. Samples collected from mice at 5, 10, and 15 months are indicated in red, green and blue respectively; those collected from female and male mice are expressed with circles and triangles respectively; those collected from mice on the CD or WD are designated as open or closed markers respectively.

Analysis of the serum metabolome through unsupervised non-metric multidimensional scaling (NMDS) is shown in Fig. 2. A clear differentiation between age (5 vs. 10 vs. 15 months), as well as differences based on sex (circle vs. triangle markers) were observed (Fig. 2a). The impact of diet could also be observed (closed vs. open markers), but only when each sex was plotted separately (Fig. 2b,c). Application of a linear model to evaluate the impact of diet, sex, and age revealed that out of 51 metabolites, 41 were significantly different by age, 31 by sex, and 23 by diet (Supplementary Table 1). Only a few metabolites had interaction effects between the variables. Significant interaction terms for age and sex were observed for 2-hydroxyisobutyrate, acetone, betaine, fumarate, and trimethylamine N-oxide (TMAO), and for diet and age were arginine, carnitine, and formate. The only metabolite that had a significant interaction term between diet and sex, as well as diet, age, and sex was TMAO.

Several metabolites tended to increase with age in all mice and included amino acids (alanine, aspartate, isoleucine, lysine, and ornithine), pyruvate and lactate, succinate, as well as choline and creatine. Metabolites that tended to decrease with age included glycerol and ketone bodies (3-hydroxybutyrate, acetoacetate, and acetone) (Supplementary Tables 1 and 2, and Supplementary Fig. 1).

Metabolite differences based on sex regardless of diet and age included 2-hydroxyisobutyrate, 2-oxoglutarate, alanine, asparagine, aspartate, glutamate, glycine, lactate, leucine, malate, phenylalanine, taurine, threonine, trimethylamine (TMA), tyrosine, and valine, which were all higher in male compared to female mice (Supplementary Tables 1 and 2). Those metabolites consistently higher in female mice regardless of diet and age included betaine and glycerol.

Among 23 serum metabolites that were significantly different with diet (Supplementary Table 1), the impact of diet was assessed for each age group for male and female mice separately (Supplementary Fig. 2). Glucose and the tricarboxylic acid (TCA) cycle intermediate 2-oxoglutarate, were higher in mice consuming the WD compared to those consuming the CD for both sexes (Supplementary Fig. 3). Interestingly, other TCA cycle intermediates, fumarate and malate trended lower in mice consuming the WD. Arginine was lower in mice on the WD at 10 and 15 months of age, whereas ornithine was higher in mice consuming the WD compared to mice consuming the CD of both sexes at all ages. Isoleucine and valine were lower in mice consuming the WD, although this difference was no longer statistically significant after application of a post-hoc test. Although threonine and tyrosine levels were not different between diets at 5 months of age, their levels were significantly lower in male mice on the WD at 10 and 15 months. Although low in concentration, serum TMA, which is derived from microbial activity, was lower in male mice on the WD at all ages. TMAO, which is derived from TMA by the action of flavin monooxygenase (FMO) in the liver, was lower in female mice on the WD at all ages. Since both TMA and TMAO are derived from the same source, we compared serum TMA + TMAO levels. TMA + TMAO were significantly higher in mice on the CD versus mice on the WD; however, no significant difference was found between sexes (Supplementary Fig. 4).

**Figure 3:**
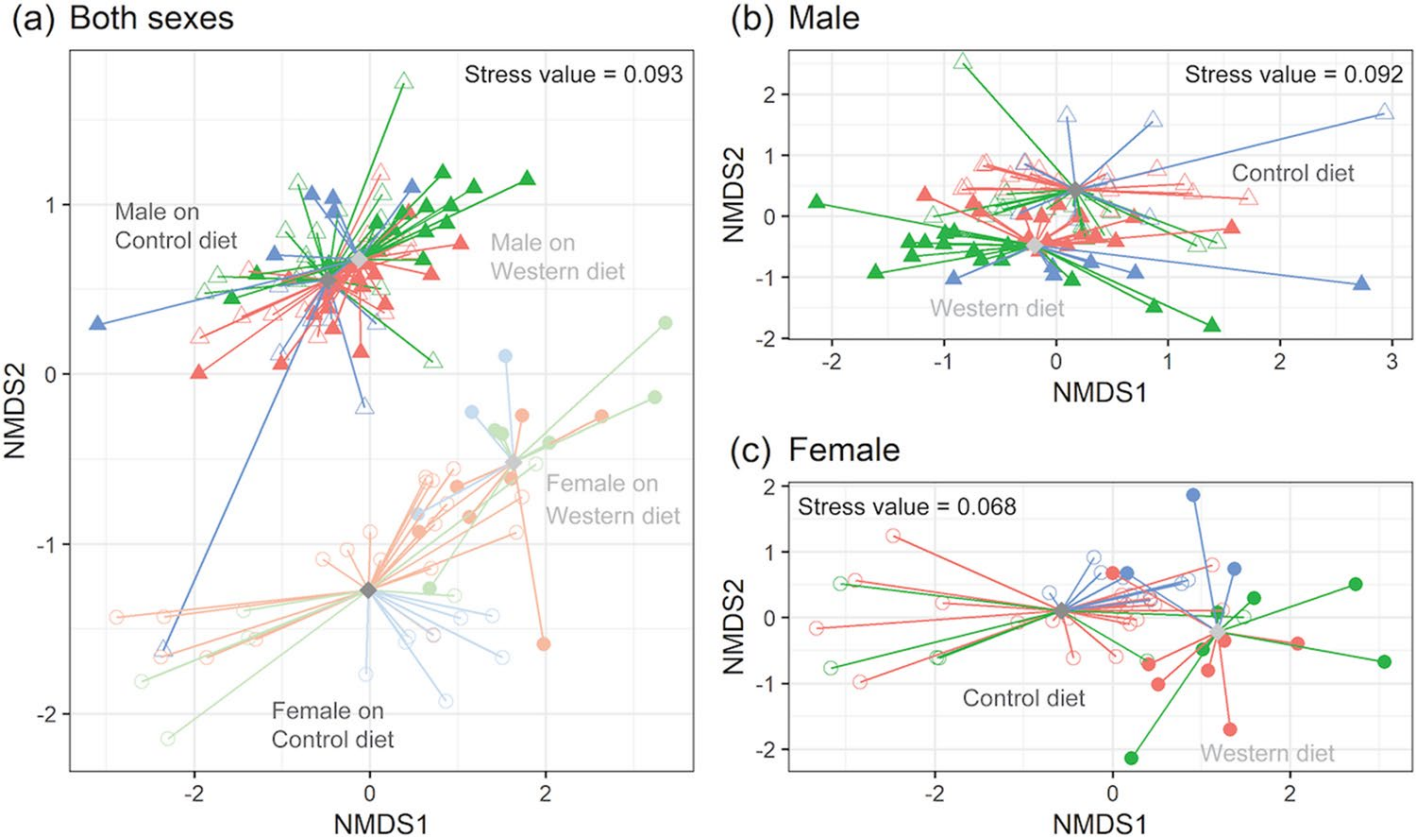
NMDS plots of the urine metabolome. The centroid of each group is noted as a diamond. (**a**) NMDS plot generated with all samples included; (**b**) NMDS plot generated with only male samples; (**c**) NMDS plot generated with only female samples. Samples collected from mice at 5, 10, and 15 months are indicated in red, green and blue respectively; those collected from female and male mice are expressed with circles and triangles respectively; those collected from mice on the CD or WD are designated as open or closed markers respectively.

**Figure 4:**
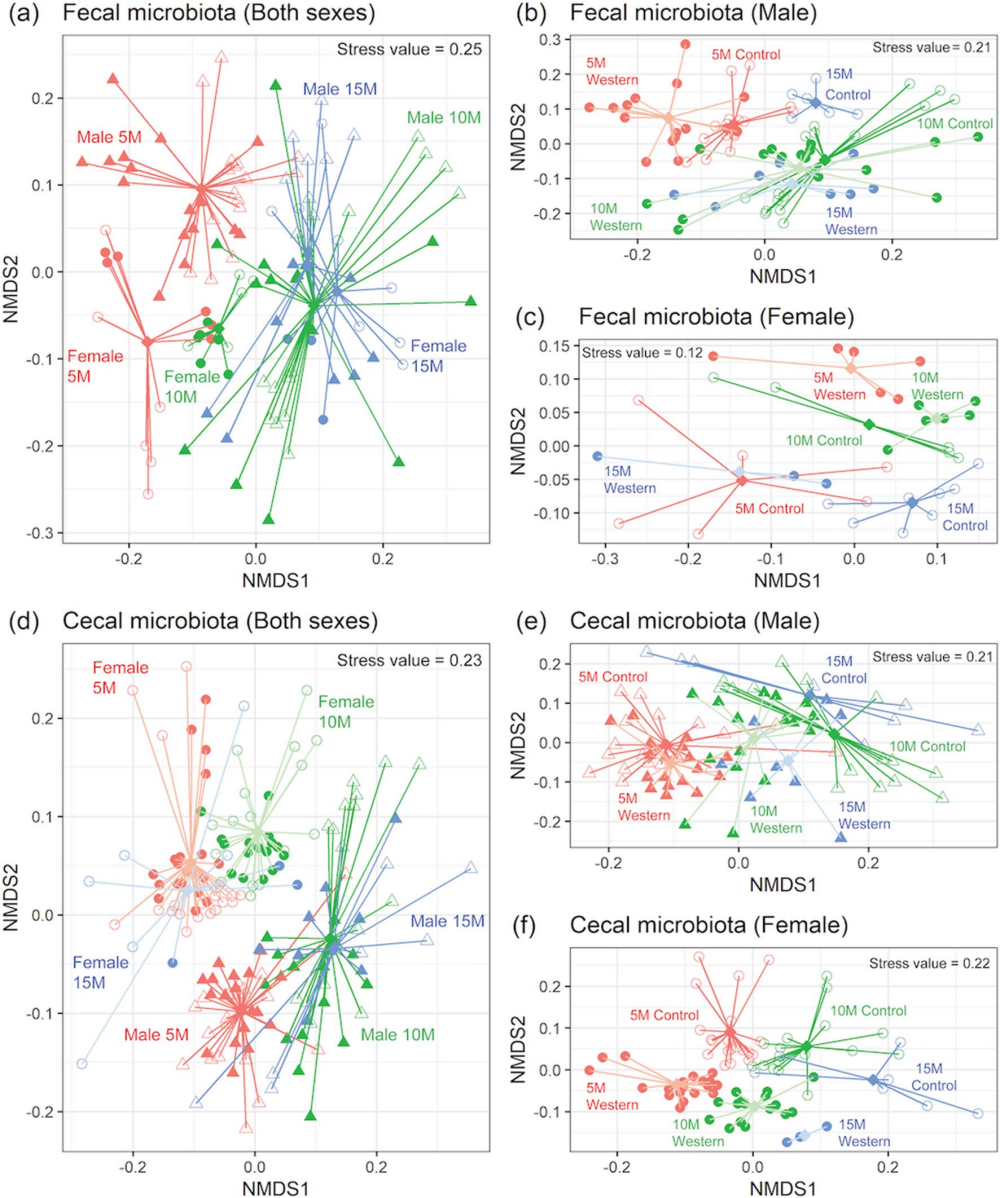
NMDS plots generated with the microbiota dataset from (**a**) fecal samples from both sexes; (**b**) fecal samples from male mice; (**c**) fecal samples from female mice; (**d**) cecal samples from both sexes; (**e**) cecal samples from male mice; and (**f**) cecal samples from female mice. The centroid of each group is noted as a diamond. Samples collected from mice at 5, 10, and 15 months are indicated in red, green and blue respectively; those collected from female and male mice are expressed with circles and triangles respectively; those collected from mice on the CD or WD are designated as open or closed markers respectively.

One weakness of our study was the fact that mice were not fasted for a defined amount of time prior to blood collection at necropsy. To determine whether this might impact our results, we compared blood glucose levels obtained in this study with a previous study analyzing blood glucose in C57BL/6J mice after 12 h fasting on either a chow diet or a high-fat diet^6^. Compared with their chow-fed male mice, our CD fed male mice had significantly higher average blood glucose at 5 (but not 10 or 15) months (91 ± 18 vs. 126 ± 27 mg/dL respectively, *p* < 0.0001). Compared with their high-fat fed mice, our WD fed mice at 5 (but not 10 or 15) months had significantly higher blood glucose (139 ± 25 vs. 170 ± 41 mg/dL respectively, *p* = 0.03). We subsequently identified those mice with blood glucose levels above the highest blood glucose level reported by Sun et al.^6^ (123 mg/dL for chow-fed mice and 171 mg/dL for high-fat fed mice), and removed them from serum metabolomics analysis at 5 months (“reduced serum dataset”). A total of 7/14 mice in CD group and 5/13 in WD group were removed. Re-analysis of serum glucose concentrations revealed slightly higher average glucose (107 mg/dL, *p* = 0.047) for CD fed mice, but WD fed mice were not significantly higher than reported by Sun et al.^6^ (145 mg/dL, *p* = 0.41). We subsequently re-applied our statistics on this reduced serum dataset and compared with the original one. No difference was found in the position of the centroids in the NMDS plots nor in the statistical results of metabolites previously reported to change with fasting^7^, such as ketone bodies, 2-hydroxybutyrate, and methionine (Supplementary Fig. 5). Moreover, when the original and reduced datasets were compared, no statistically significant difference was found for any of the metabolites.

**Figure 5:**
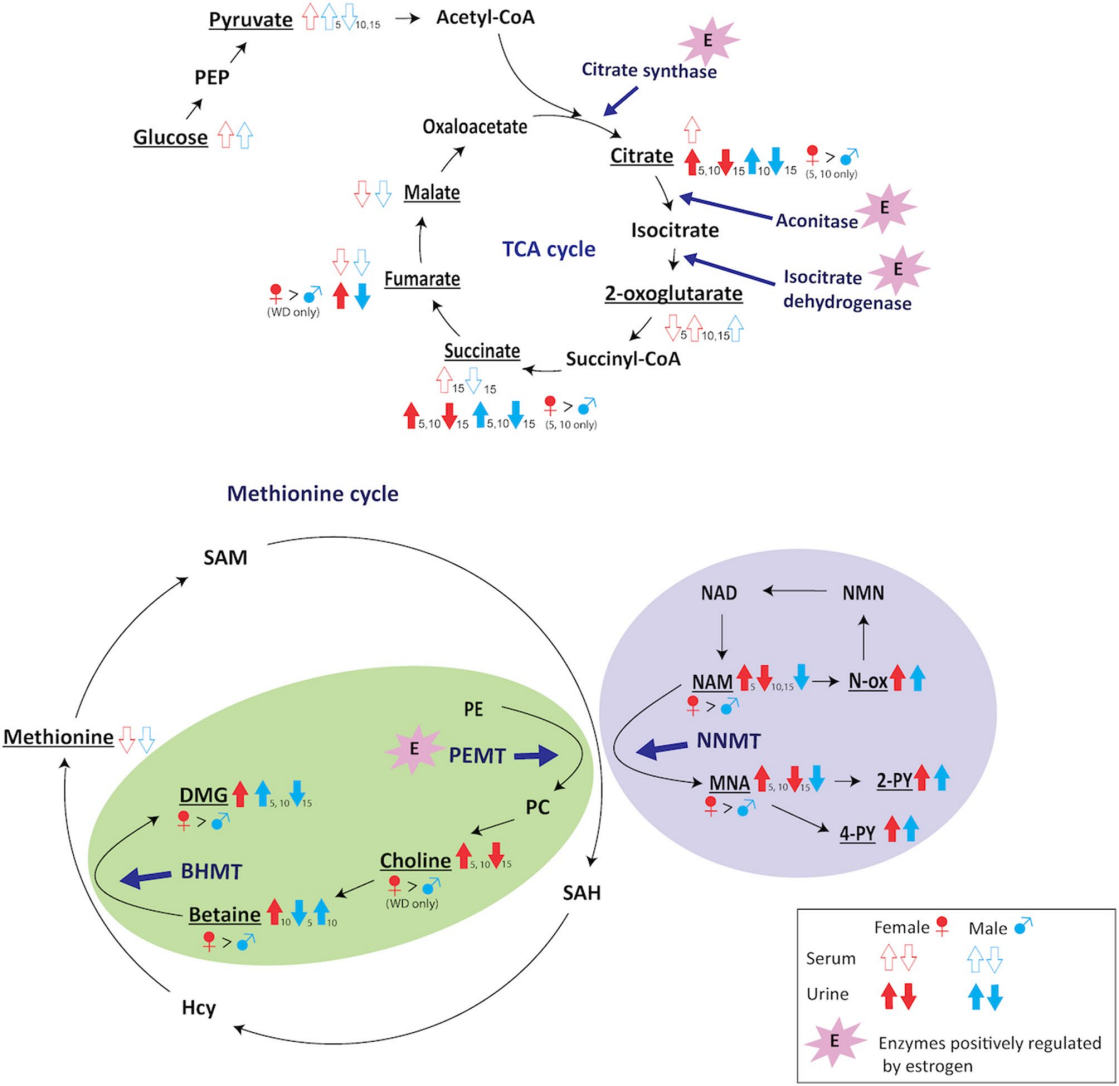
Schematic depiction of the metabolites involved in PEMT, BHMT, and NNMT pathways, and the TCA cycle. The arrows represent the trajectory of median concentrations of serum (open arrows) or urinary (closed arrows) metabolites in mice on the WD compared to those on the CD. The numbers next to the arrows represent the age of the mice at sample collection. Males are represented in blue and females in red. Differences in median metabolite concentrations between the two diets larger than 10% are included, with metabolites profiled in this study underlined. Further trends and statistical significance can be found in Supplementary Figs. 1 and 3. Enzymes positively regulated by estrogen are noted.

### The urine metabolome was influenced primarily by sex, and less by diet

Analysis of the urine metabolome through unsupervised NMDS is shown in Fig. 3. Here, separation was greatest based on sex (Fig. 3a), with differences in diet more apparent when male and female mice were plotted separately (Fig. 3b,c). Through application of a linear model to evaluate the impact of diet, sex and age, we observed that out of 53 metabolites, 38 were significantly different by sex, 30 by diet, and 24 by age (Supplementary Table 3). Interestingly, 34 metabolites exhibited a significant interaction term between diet and sex, 30 between age and sex, 22 between diet and age, and 1 between diet, age, and sex. Of interest, those metabolites with significant interaction terms between diet and sex or age and sex were metabolites associated with gut microbiota (including 2-hydroxyisobutyrate, 3-indoxylsulfate, 4-hydroxyphenyllactate, N-phenylacetylglycine, TMA, TMAO), as well as nicotinamide metabolism (N-methyl-2-pyridone-5-carboxamide (2PY), N-methyl-4-pyridone-5-carboxamide (4PY)). In contrast, most metabolites with significant interaction terms between diet and age were related to endogenous metabolism.

Metabolites higher in female mice included those involved in nicotinamide metabolism (niacinamide (NAM) and 1-methylnicotinamide (MNA)) (Supplementary Table 4 and Supplementary Fig. 6). These were ~ 3 times higher in female compared to male mice at 5 and 15 months. Although similar in concentration between the sexes at each age and under each dietary condition, consumption of the WD led to significant elevations of 2PY and 4PY at 5 and 10 months compared to mice on the CD (Supplementary Fig. 3). The TCA cycle intermediates citrate and succinate were higher in females compared to males on both diets at 5 and 10 months, with the sex difference greater in mice on the WD. Fumarate followed the same trend, but only in mice on the WD. In female mice, although citrate and succinate levels were significantly higher in the WD group at 5 and 10 months of age, their levels dropped at 15 months of age, with citrate significantly lower in the WD group. Interestingly, we found that choline was significantly higher in female mice on the WD at 5 and 10 months of age, and betaine and DMG were significantly higher regardless of age and diet compared to male mice, which dropped to levels more similar to male mice at 15 months of age (Supplementary Fig. 1).

Metabolites derived from microbial activity, including 2-hydroxyvalerate and 4-hydroxyphenylacetate, were higher in male compared to female mice on the CD. These metabolites were not significantly different when comparing WD-fed mice. On the other hand, acetate, hippurate, and isobutyrate were lower in male mice on either diet. Both 3-indoxyl sulfate and N-phenylacetylglycine trended higher in the urine of female mice compared to male mice only in mice consuming the WD. Interestingly, although male mice showed significantly higher levels of TMA and lower levels of TMAO compared to female mice on either diet, the significant sex differences disappeared in mice on the WD at 15 months of age (Supplementary Fig. 1). Comparison of urinary TMA + TMAO levels revealed that they were significantly higher in mice on the CD versus mice on the WD, and further that they were consistently and significantly higher in male mice compared to female mice on the CD at 5 and 10 months (Supplementary Fig. 4).

Although sex and diet were the main factors that affected the urinary metabolome, the impact of age was reflected in trends toward increased TMA and decreased 4-hydroxyphenylacetate, ascorbate, and DMG with age (Supplementary Tables 3 and 4).

### Fecal and cecal gut microbiota were impacted primarily by sex, which was also reflected in the urine metabolome

It is known that some metabolites in serum and urine are derived by the gut microbiota-host interactions. To further understand the impact of sex, diet and age on the gut microbiome, we evaluated the 16S rRNA gene sequences of feces and cecal content in male and female mice on a CD or WD at 5, 10, and 15 months of age (Fig. 4). Fecal microbial profiles visualized by NMDS plots revealed the clearest separation for age and sex (Fig. 4a), with the impact of diet more apparent when male and female samples were plotted separately (Fig. 4b,c). NMDS analysis of cecal microbiota revealed a large impact with sex, followed by diet (Fig. 4d–f). Application of multivariate response linear regression on the microbial communities to evaluate the effects of diet and sex at each time point (Supplementary Table 5) revealed the variation in the microbial composition explained by diet and sex were 29.4, 23.3, and 33.5% for fecal samples, and 28.8, 22.9, and 37.9% for cecal samples at 5, 10, and 15 months of age, respectively. To estimate the effect of a single covariate, a leave-one-variable-out approach was used, and the results reported as “R^2^diff”. Interestingly, a larger percentage of the microbial variables were explained by sex rather than diet at all time points for both fecal and cecal samples. Microbes that were identified to be significantly different between sex or diet by ordinary least squares (OLS) regression are summarized in Supplementary Tables 6 and 7 for fecal and Supplementary Tables 8 and 9 for cecal microbiota, respectively.

With aging, a steady shift was found in the relative abundance of fecal *Allobaculum* (increasing in male mice), *Dorea* (decreasing in female mice), and *Ruminococcus* (decreasing in female mice on the WD), *Erysipelotrichaceae* (decreasing in female and increasing in male mice) (Supplementary Tables 6 and 7, Supplementary Fig. 4). Between sexes, the relative abundance of fecal *Allobaculum* and *Erysipelotrichace* were higher in male mice, whereas *Dorea* and *Ruminococcus* were higher in female mice.

For cecal microbiota, generally *Allobaculum* was observed to be higher in male mice on either diet, *Anaeroplasma* was higher in male mice on the CD, *Dorea* was higher in female mice on either diet, *Erysipelotrichaceae* was higher in male mice on either diet, and *Anaeroplasmataceae* was higher in male mice on the CD (Supplementary Tables 8 and 9, Supplementary Fig. 4). Of interest, application of a Spearman correlation revealed a significant correlation between both serum and urine TMA levels and fecal *Erysipelotrichaceae* (Supplementary Fig. 4).

## Discussion

Obesity is the consequence of excess energy accumulation due to a chronic imbalance of energy intake over energy expenditure. Our view on this energy imbalance is guided by the multitude of studies that have used only male subjects in investigating the impact of diet-induced obesity on health^8,9^. It is established that energy metabolism is different in females due to sexual dimorphism in the levels of hormones, fat distribution^9^, and mitochondrial function^10^. However, few studies have examined these differences at different life stages. Our study aimed to elucidate sex differences in the metabolome in response to a WD consisting of excessive fat (42% kcal) and sucrose (43% kcal) (Supplementary Table 10). A unique aspect of the current study was to understand the development of obesity over time, from early adulthood (5 months in a mouse, which corresponds to roughly 20–30 years of age in a human) to mature adulthood (15 months in a mouse, which corresponds to ~ 50 years old in a human)^11^. This enabled us to capture the dynamic changes in the metabolome with aging in both male and female mice, which is not possible with common study designs that look at the consequence of diet-induced obesity at a single timepoint. The gut microbiota was also of interest as it has been associated with changes in host energy intake, lipid metabolism, oxidation, and hormone production, which may affect host adiposity and the risk of obesity and metabolic disorders^4, 5^.

Consumption of the WD induced profound changes in metabolism in both male and female mice. Significant elevation in urinary 2PY and 4PY with consumption of the WD suggest increased activity of the enzyme nicotinamide N-methyltransferase (NNMT) (Fig. 5). NNMT transfers a methyl group from S-adenosylmethionine (SAM) to S-adenosylhomocysteine (SAH) creating MNA that is oxidized by aldehyde oxidase to 2PY and 4PY, which are subsequently excreted in urine^12^. Elevated activity of NNMT in white adipose tissue has been previously reported in C57BL/6 mice on a high-fat diet^13^, and has been shown to be a risk factor for development of non-alcoholic steatohepatitis (NASH)^14^. Knockdown of NNMT has been reported to have protective effects against diet-induced obesity, including lowering fat mass, serum triglycerides, free fatty acids, as well as increasing insulin sensitivity in mice with elevated energy consumption^12^. While NNMT activity is likely higher in mice on the WD, we also observed higher urinary MNA levels in female compared with male mice. Higher MNA has been associated with increased expression levels of Sirtuin 1 (Sirt1) in vivo^15^. Sirt1 upregulates hepatic gluconeogenesis^16^ and ameliorates hepatic lipid accumulation as well as muscle mitochondrial biogenesis^15^. It is possible that increased MNA in females might be an adaptive mechanism against the high-fat diet and could possibly explain the reported decrease in risk for non-alcoholic fatty liver disease (NAFLD) in females compared to males^17,18^.

One of the important sex differences in response to the WD was a significantly higher level of urinary choline in female mice on the WD. Since dietary intake of choline was the same for all mice, the difference in choline might be due to upregulation of phosphatidylethanolamine N-methyltransferase (PEMT)^19^. PEMT catalyzes methylation of phosphatidylethanolamine (PE) to produce phosphatidylcholine (PC)^20^, which is subsequently converted to choline (Fig. 5). Choline can be used to synthesize PC or oxidized to betaine in the liver and kidney^21^. Betaine is converted to DMG by betaine-homocysteine methyltransferase (BHMT)^22^. Both betaine and DMG were significantly higher in female compared to male mice on either diet, but the difference was larger in response to the WD, suggesting that BHMT activity is higher in females, and is elevated in response to the WD. Importantly, the levels of these metabolites decreased in female mice at 15 months of age. Considering that PEMT is positively regulated by estrogen^19^, a decline in PEMT could potentially be explained by an age-related reduction in estrogen. Indeed, female mice show reproductive senescence between 9 and 12 months of age^23^, and thus female mice in our study would likely have had a reduction in estrogen at the age of 15 months. This is supported by the drop in urinary TCA cycle intermediate levels in 15-month old female mice on the WD. Estrogen upregulates enzymes in the early part of the TCA cycle (citrate synthase, aconitase, and isocitrate dehydrogenase), but not those involved in the latter part of the cycle^24^ (Fig. 5). A reduction in estrogen may explain why female mice on the WD showed a significantly higher rate of weight gain than male mice at 15 months. Indeed, the reduction of estrogen in post-menopausal women has also been associated with higher weight gain^25^. The impact of estrogen on the PEMT pathway may be protective for females prior to reproductive senescence. High plasma homocysteine has been reported in obese populations^8^, which is associated with complications such as placental abruption or infarction during pregnancy^26^. Considering that in response to high-fat diet, mRNA expression of BHMT^27^ as well as hepatic BHMT activity were elevated together with lower SAM and higher SAH levels^8^, female mice on WD may have reacted to over-nutrition by elevating PEMT and BHMT to dispose of elevated homocysteine. These protective mechanisms may be important to store fat and prepare for famine, as well as to make milk for the offspring as it has been shown that 70% of breast milk linoleic acid is derived from maternal body fat stores^28^. Indeed, prior to menopause, females have a lower risk of cardiovascular disease, and higher levels of homocysteine has been associated with an increased risk of heart disease^29^.

The complex interaction between the impact of diet, sex, and age was also observed with microbial metabolites both in the serum and urine metabolomes. For example, TMA and TMAO were significantly different based on sex and diet in both serum and urine samples, and microbes associated with TMA generation (*Erysipelotrichaceae*^30^) were positively correlated with serum and urine TMA. In agreement with a previous study^31^, we observed that male mice had higher TMA but lower TMAO levels in both the serum and urine compared to female mice. This is likely due to different expression levels of flavin monooxygenases (FMOs). TMAO is generated by conversion of absorbed TMA to TMAO by FMO enzymes in the liver^32^. While female mice have activities of FMO1, FMO3, as well as FMO5 in the liver, male mice suppress activity of hepatic FMO3 at 5–6 weeks of age due to sex steroids^32^. There are mixed results regarding the impact of a high-fat diet on TMA/TMAO metabolism. Some studies have reported an elevated level of circulating TMAO in mice on a high-fat diet^33^, which was associated with cardiovascular disease^34^. Others have shown a reduction in the serum^35^ or urinary^36^ TMAO level in mice fed high-fat diet, which is concordant with what we found. The discrepancies among studies may come from the fact that TMA is generated by microbial activity, and could be dependent on initial colonization.

Another metabolite derived from gut microbiota, 3-indoxyl sulfate (3-IS), was higher in female urine on the WD compared with male mice. Operational taxonomic units (OTUs) associated with higher urinary 3-IS are members of *Ruminococcaceae*^37^, and indeed, our study revealed that female mice had higher relative abundance of fecal *Ruminococcus* on either diet. 3-IS is derived from gut microbial metabolism of tryptophan by gut microbes^37^. Although 3-IS has been recognized as a uremic toxin and a marker of renal disease, some studies revealed it could have positive effects^38^. For example, Weber et al.^37^ analyzed 3-IS levels in patients undergoing allogeneic stem cell transplantation, which is often associated with severe complications due to disruption of the gut microbiome*.* Higher level of urinary 3-IS was associated with lower mortality rate as well as protection from inflammation by alteration of pro- and anti-inflammatory cytokines^37^. Another gut microbial metabolite, hippurate, was found to be higher in urine samples from female mice compared to male mice on either diet. Such differences have been previously reported in rats, mice, as well as in humans^39^. Although hippurate was found to be lower in obese animals in previous studies^39^, here we found mice consuming the WD had increased urinary levels. These results highlight how some microbial markers provide clues about microbial activity, but it is clear that much work remains to understand the significance in terms of health outcomes.

In both males and females, the impacts of age on serum lactate and ketone bodies were similar. Plasma lactate has been shown to increase in the brain with aging, and may constitute an alternate energy source^40^, since aging results in a lower abundance of the glucose transporter GLUT1 in the brain, and increased abundance of monocarboxylate transporter 1 (MCT1)^41,42^. The increase in MCT1 in the brain may increase ketone body uptake^43^ to counteract the age-related decrease in ketogenesis in the liver^44^.

To our knowledge, this is the first study to comprehensively investigate the impact of aging and diet in male and female mice. Limitations of this study include that we were not able to conduct metabolomics and microbiota analyses at baseline before dietary challenges were introduced, and that estrogen levels were not measured due to sample limitations. Other limitations include a lack of uniform fasting time and small sample size for certain measurements. Nonetheless, we demonstrated that the serum and urine metabolomes and gut microbiota of male and female mice have some similarities and differences with diet. Our results highlight that significant metabolic differences exist with age and sex, and that these factors need to be considered when generalizing the impact of diet on metabolism.

## Methods

### Animals and design

A total of 120 male and female C57BL/6J wild-type (Jackson Laboratory) mice were purchased at 3 weeks of age, and were subsequently housed in an air-conditioned room with 12 h light/dark illumination cycles cycling on at 6 am and off at 6 pm at a constant temperature and humidity. Half of the mice were given a Western diet (WD; 21.2% fat, 34% sucrose, and 0.2% cholesterol by weight), and the other half a regular chow diet as the control diet (CD; 5.2% fat, 12% sucrose, and 0.01% cholesterol by weight) purchased from Harlan Teklad from the age of 3 weeks. The formula, nutritional composition, and fatty acid (FA) profiles of CD and WD used in this study are summarized in Supplementary Tables 10, 11, and 12, respectively. WD contained higher g/kg saturated FAs (palmitic acid and stearic acid), monounsaturated FAs (oleic acid), and trans FAs, whereas CD contained higher levels of polyunsaturated FAs (linoleic acid and linolenic acid). WD contained 34% sucrose whereas CD contained 12% sucrose by weight with 68% and 13% of kcal from carbohydrate and fat respectively for CD, and 43% and 42% of kcal from carbohydrate and fat respectively in WD.

All mice were supplied their respective diets with water ad libitum. At the age of 5, 10, or 15 months, urine and fecal samples were collected from one third of the mice, followed by serum and cecal samples at necropsy. As is common with C57BL/6 mice, several mice (particularly female mice on WD) contracted ulcerative dermatitis^45^ severely restricting the sample sizes at 15 months. Not all mice produced a fecal or urine sample at each time point. After necropsy, in some cases not enough blood or cecal sample could be collected for analysis, and thus sample size varied for cecal and blood samples. Sample sizes for each sample type by diet, time point, and sex is provided in Supplementary Table 13. On the day of necropsy, male mice were sacrificed between 9:00 and 9:45 am across 4 consecutive days and female mice were sacrificed between 10:30 am and 12 pm across 2 consecutive days. Although food was not taken away from the animals at a set time prior to sacrifice, in general the mice did not eat after 6 am when the lights were turned on. Protocols were approved by the Institutional Animal Care and Use Committee of the University of California, Davis and were conducted following National Institutes of Health Guide for the Care and Use of Laboratory Animals.

### Metabolomics analysis

For metabolite extraction, serum and urine samples were thawed on ice and filtered by Amicon Ultra-0.5 mL centrifugal filter (3 k molecular weight cutoff, Millopore, Billerica, MA) to remove protein and lipid particles. 207 µL of the filtrate was mixed with 23 µL of an internal standard (Chenomx Inc., Edmonton, Alberta, Canada) containing 4.608 mM 3-(trimethylsilyl)-1-propanesulfonic acid-d 6 (DSS-d6) and 0.2% NaN_3_ in 98% D_2_O. Samples were stored at 4 $^\circ{\rm C}$ overnight, and the pH was adjusted to 6.85 ± 0.07 for each sample by adding a small volume of 1 N HCl or NaOH. 180 µL of each sample was transferred into a 3 mm NMR tube and samples were kept at 4 $^\circ{\rm C}$ until NMR data acquisition within 24 h of sample preparation. All ^1^H NMR spectra were acquired at 25 $^\circ{\rm C}$ using the noesypr1d pulse sequence on an Avance 600 MHz NMR spectrometer (Bruker, Billerica, MA) equipped with a SampleJet as previously described^46^. Identification and quantification of metabolites (μM) were completed using Chenomx NMRSuite (version 8.1, Chenomx Inc., Edmonton, Canada)^47^.

### Microbiome analysis

Cecal and fecal samples were stored at -80 $^\circ{\rm C}$ until analysis. DNA was extracted from 100 mg of samples using ZR Fecal DNA MiniPrep Kit (Zymo Research), quantified by the NanoDrop (NanoDrop Technologies), and amplified with the primers as described previously^48^. Illumina MiSeq sequencing of variable region 4 (V4) of 16S ribosomal RNA was run following the published methods^49,50^. Raw 16S rRNA high throughput sequencing data from each of the three sequencing runs was demultiplexed using sabre (https://github.com/najoshi/sabre). Demultiplexed reads were quality filtered and dereplicated in QIIME2 with the DADA2 plugin^51,52^. Ribosomal sequence variants (RSVs) with fewer than 10 total reads and RSVs in five or fewer samples were filtered from the DADA2 feature table and representative sequence files.

### Statistics

All statistical analyses were conducted in R (version 3.5.1) and Benjamini–Hochberg FDR correction was applied to all* p* values obtained for multiple comparisons. NMDS plots were generated using metaMDS in the vegan package (version 2.5.2) of R using the ggplot2 package (version 3.0.0). A Euclidean distance was used for metabolome analysis, whereas the Bray Curtis function was used for analysis of microbiota data. Metabolite contributions to clustering were assessed using envfit.

Weight gain rate (%) was obtained by dividing the weight at either 5, 10, or 15 months old by the weight at baseline (3 weeks old) for each animal. Prior to applying statistics, serum and urine concentrations were log-transformed and tested for normality using the Shapiro–Wilk test. Metabolites with *p* > 0.05 and/or W > 0.80 were considered to have normal distribution. For metabolites that did not satisfy the normal distribution criteria, qqplots were generated to identify outliers. All metabolites except for serum asparagine, citrate, and N-methylhydantoin met the normality criteria. For these serum metabolites, one female sample at 5 months of age and one male sample at 10 months of age were removed as outliers (Supplementary Fig. 5). All metabolites profiled in the urine sample satisfied the criteria of normal distribution, so no samples were removed as outliers. A linear model was fitted and then ANOVA was applied to evaluate the impact of diet sex and age on the serum and urine metabolites. As the post-hoc test, estimated marginal means were obtained using emmeans (version 1.2.3) and pairwise comparison was applied.

From the NMDS plot on the cecal microbiota, one sample from a female mouse on CD collected at 10 months of age was identified as an outlier and removed in order to better observe the distribution (Supplementary Fig. 5). Differential abundance analysis was performed using gneiss, which is a compositional data analysis tool that uses a concept known as “balance trees”, in combination with OLS regression models to identify taxa significantly different between groups (a cut-off* p* value of 0.01 after Bonferroni correction was used)^53^. A pseudocount of 1 was added to the values of the filtered DADA2 sequence table, followed by the application of Ward’s hierarchical clustering to identify microbes that co-occur, or the “Principal Balances”. These were then transformed with the isometric log-ratio calculation. Once the balances were computed, ordinary least square (OLS) regression was applied to evaluate the effect of sex and diet on the microbial community. And a leave-one-variable-out approach was used to estimate the effects of a single covariate. Pearson correlation was used to assess the correlation between metabolite concentration and microbial relative abundance, and significant correlation was defined with a* p* value of less than 0.05 and a higher than medium effect size (estimated measure of association, R > 0.3^54^) were found.

## Supplementary information

Supplementary Information.

## Abbreviations

SAM: S-adenosylmethionine
SAH: S-Adenosyl-L-homocysteine
Hcy: Homocysteine
PE: Phosphatidylethanolamine
PEMT: Phosphatidylethanolamine N-methyltransferase
PC: Phosphatidylcholine
DMG: N,N-dimethylglycine
BHMT: Betaine-homocysteine methyltransferase
NAD: Nicotinamide adenine dinucleotide
NMN: Nicotinamide mononucleotide
NAM: Nicotinamide
N-ox: Nicotinamide N-oxide
NNMT: Nicotinamide N-methyltransferase
MNA: 1-Methylnicotinamide
2PY: N-methyl-2-pyridone-5-carboxamide
4PY: N-methyl-4-pyridone-5-carboxamide
PEP: Phosphoenolpyruvate
TCA cycle: Tricarboxylic acid cycle
