# Supplementary material for: Long-term effects of western diet consumption in male and female mice
Source: Sci Rep. 2020 Sep 7;10:14686. doi: 10.1038/s41598-020-71592-9 (PMC7477228; doi:10.1038/s41598-020-71592-9)
Supplement: Supplementary file 1 — Supplementary Information. [file 41598_2020_71592_MOESM1_ESM.pdf]

Supplementary information

Long-Term Effects of Western Diet Consumption in Male and Female Mice

Yu Hasegawa, Shin-Yu Chen, Lili Sheng, Prasant Kumar Jena, Karen M. Kalanetra,

David A. Mills, Yu-Jui Yvonne Wan, Carolyn M. Slupsky*

Table of contents

- **Supplementary Tables**
    - **Supplementary Table 1.** Summary of linear regression analysis to evaluate the impact of diet, sex and age on serum metabolites.
    - **Supplementary Table 2.** Serum metabolite concentrations (μmol/L) from male and female mice on a standard chow control diet (CD) or Western diet (WD).
    - **Supplementary Table 3.** Summary of linear regression analysis to evaluate the impact of diet, sex and age on urine metabolites.
    - **Supplementary Table 4.** Urine metabolite concentrations (μmol/L) from male and female mice on the CD or WD.
    - **Supplementary Table 5.** Summary of multivariate response linear regression analysis on the balance of microbial communities.
    - **Supplementary Table 6.** Fecal microbes identified as significantly different in abundance between male and female mice accounting for diet.
    - **Supplementary Table 7.** Fecal microbes significantly in abundance between CD and WD accounting for sex.
    - **Supplementary Table 8.** Cecal microbes significantly different in abundance between male and female mice accounting for diet.
    - **Supplementary Table 9.** Cecal microbes significantly different in abundance between CD and WD accounting for sex.
    - **Supplementary Table 10.** Nutritional composition of the diets used in this study.
    - **Supplementary Table 11.** Formula of the diets used in this study.
    - **Supplementary Table 12.** Fatty acid composition of the diets used in this study.

- **Supplementary Table 13.** Sample sizes for serum, urine, fecal and cecal samples.

Supplementary Figures

- **Supplementary Figure 1.** Boxplots showing the concentrations of serum and urine metabolites.
- **Supplementary Figure 2.** Volcano plots showing the difference in the serum metabolome between CD and WD for male and female mice.
- **Supplementary Figure 3.** Heatmaps showing the rate of increase in the median concentration of metabolites from the WD group compared to the CD group.
- **Supplementary Figure 4.** Boxplots showing the concentrations of TMA+TMAO measured from serum and urine samples as well as the relative abundance of fecal and cecal gut microbes. Correlation between fecal abundance of *Erysipelotrichaceae* and serum or urinary TMA.
- **Supplementary Figure 5.** NMDS plots before and after removing samples with high glucose.
- **Supplementary Figure 6.** Volcano plots showing the difference in the urine metabolome between male and female mice on either the CD or WD.
- **Supplementary Figure 7.** Outliers removed in this study from the serum and cecal datasets.

Supplementary Table 1. Summary of linear regression analysis to evaluate the impact of diet, sex and age on serum metabolites. Metabolites with $p < 0.05$ after FDR correction for at least one factor were included in the table, and p-values for each term are provided.

Metabolite	Diet	Age	Sex	Diet:Age	Diet:Sex	Age:Sex	Diet:Age:Sex		Metabolite	Diet	Age	Sex	Diet:Age	Diet:Sex	Age:Sex	Diet:Age:Sex
1,3-Dihydroxyacetone	3.24E-01	5.16E-28	4.79E-01	7.86E-01	2.93E-01	6.83E-01	8.44E-01		Glycine	2.60E-01	2.17E-02	1.56E-03	9.56E-01	6.36E-01	9.42E-01	9.69E-01
2-Hydroxybutyrate	1.39E-02	1.37E-01	2.15E-01	5.84E-01	7.01E-01	3.84E-01	8.58E-01		Glycolate	1.02E-02	6.55E-11	4.16E-01	4.08E-01	2.93E-01	4.67E-01	9.29E-01
2-Hydroxyisobutyrate	1.05E-06	5.96E-05	4.59E-36	9.64E-01	1.41E-01	6.20E-03	8.44E-01		Histidine	3.23E-01	2.78E-02	6.75E-01	9.56E-01	8.52E-01	7.30E-01	8.44E-01
2-Oxoglutarate	3.92E-03	3.08E-04	8.49E-03	6.63E-02	4.34E-01	7.30E-01	9.69E-01		Isoleucine	3.25E-02	3.04E-04	1.11E-01	8.11E-01	7.27E-01	7.32E-01	8.58E-01
2-Oxoisocaproate	6.31E-01	3.37E-05	7.17E-01	8.24E-02	3.75E-01	7.18E-01	5.92E-01		Lactate	1.21E-02	1.00E-15	4.93E-07	4.08E-01	7.27E-01	6.83E-01	6.09E-01
3-Hydroxybutyrate	7.96E-02	1.54E-04	7.09E-03	7.40E-01	9.57E-01	9.43E-01	9.45E-01		Leucine	2.65E-01	1.72E-04	1.64E-04	8.11E-01	9.47E-01	6.83E-01	9.69E-01
3-Hydroxyisobutyrate	5.17E-08	1.73E-11	5.10E-01	8.24E-02	3.15E-01	5.02E-01	2.43E-01		Lysine	1.81E-06	3.62E-02	9.42E-01	7.86E-01	7.01E-01	9.43E-01	8.99E-01
Acetate	2.55E-01	1.49E-02	1.62E-02	8.49E-01	5.47E-01	4.67E-01	7.08E-01		Malate	2.34E-03	1.71E-01	5.46E-04	5.84E-01	1.63E-01	4.67E-01	7.08E-01
Acetoacetate	1.45E-01	7.13E-10	2.26E-03	7.86E-01	7.27E-01	7.18E-01	6.34E-01		Methionine	6.21E-04	4.33E-01	2.22E-01	5.84E-01	8.61E-01	7.32E-01	8.44E-01
Acetone	2.43E-01	3.60E-17	4.24E-01	1.16E-01	6.36E-01	2.86E-02	8.58E-01		N-Methylhydantoin	1.11E-16	6.41E-01	5.65E-08	1.54E-01	1.41E-01	4.67E-01	9.69E-01
Alanine	4.95E-01	6.01E-08	2.26E-03	2.52E-01	7.80E-01	9.43E-01	9.22E-01		O-Acetylcarnitine	4.48E-01	1.51E-02	7.09E-03	7.40E-01	7.27E-01	7.30E-01	9.69E-01
Arginine	6.21E-04	2.81E-04	1.73E-01	1.83E-05	6.36E-01	1.84E-01	8.99E-01		Ornithine	1.40E-09	2.99E-17	7.96E-02	6.63E-02	8.43E-01	1.45E-01	2.22E-01
Asparagine	6.31E-01	1.61E-04	1.70E-03	7.40E-01	7.27E-01	4.19E-01	9.69E-01		Phenylalanine	5.76E-01	2.86E-03	7.55E-05	5.45E-01	9.81E-01	9.43E-01	9.22E-01
Aspartate	1.97E-01	2.09E-09	3.39E-11	5.84E-01	7.27E-01	4.31E-01	8.19E-01		Proline	2.96E-02	1.54E-04	1.17E-02	2.52E-01	6.36E-01	7.18E-01	6.09E-01
Betaine	5.53E-01	2.08E-04	1.44E-07	2.86E-01	3.09E-01	6.20E-03	2.43E-01		Pyruvate	9.99E-01	3.33E-22	8.82E-01	6.63E-02	6.71E-02	4.67E-01	3.77E-01
Carnitine	4.95E-01	1.37E-03	1.51E-02	2.96E-02	3.09E-01	2.08E-01	7.10E-01		Serine	3.23E-01	1.66E-03	2.61E-02	8.11E-01	3.15E-01	9.43E-01	8.58E-01
Choline	1.62E-01	4.17E-15	4.26E-02	6.66E-02	7.80E-01	2.91E-01	7.10E-01		Succinate	1.45E-01	7.05E-08	2.52E-01	5.84E-01	6.36E-01	9.43E-01	8.99E-01
Citrate	1.78E-01	1.64E-03	4.24E-01	5.84E-01	2.93E-01	5.79E-02	9.22E-01		Taurine	3.23E-01	4.45E-11	5.65E-08	8.24E-02	7.27E-01	9.43E-01	7.10E-01
Creatine	6.77E-01	1.10E-03	9.03E-01	3.89E-01	7.27E-01	3.84E-01	5.20E-01		Threonine	7.03E-03	1.98E-01	1.44E-02	5.45E-01	6.36E-01	7.32E-01	8.92E-01
Ethanol (contaminant)	8.35E-01	1.10E-06	1.82E-01	1.90E-03	1.41E-01	2.28E-12	6.09E-01		Trimethylamine	6.29E-18	3.66E-03	2.05E-19	4.49E-01	8.26E-02	1.58E-01	2.22E-01
Formate	8.12E-01	8.16E-01	1.18E-01	5.61E-03	6.71E-02	1.84E-01	8.58E-01		Trimethylamine N-oxide	6.74E-04	6.51E-01	1.43E-07	4.08E-01	3.65E-06	1.13E-04	1.20E-02
Fumarate	9.15E-06	7.40E-01	3.66E-02	7.86E-01	9.47E-01	4.97E-03	9.69E-01		Tryptophan	4.36E-01	4.97E-05	1.41E-01	8.90E-01	9.81E-01	4.67E-01	7.08E-01
Glucose	2.27E-05	7.34E-10	6.78E-01	4.71E-01	6.36E-01	1.58E-01	7.10E-01		Tyrosine	2.67E-05	6.41E-02	2.71E-04	5.45E-01	6.36E-01	9.72E-01	7.10E-01
Glutamate	2.55E-01	3.67E-03	3.38E-10	8.24E-02	3.15E-01	9.43E-01	9.69E-01		Urea	1.21E-02	1.50E-02	3.18E-02	9.64E-01	6.36E-01	7.32E-01	5.92E-01
Glutamine	4.85E-02	8.08E-02	1.72E-03	8.09E-02	3.75E-01	1.58E-01	7.08E-01		Valine	2.47E-03	3.32E-03	2.06E-03	9.56E-01	7.27E-01	7.32E-01	8.58E-01
Glycerol	4.78E-01	2.96E-13	5.23E-06	6.63E-02	3.75E-01	7.32E-01	8.98E-01									

Supplementary Table 2. Serum metabolite concentrations (μmol/L) from male and female mice on a standard chow control diet (CD) or Western diet (WD). The concentrations are expressed with mean ± standard deviation (SD).

Metabolite	Sex	Control Diet									Western Diet								
		5 months			10 months			15 months			5 months			10 months			15 months		
1,3-Dihydroxyacetone	Male	183	±	84	136	±	59	14	±	12	189	±	95	147	±	87	12	±	5
	Female	201	±	43	183	±	83	20	±	10	165	±	64	82	±	51	15	±	4
2-Hydroxybutyrate	Male	16	±	4	16	±	4	15	±	3	15	±	10	13	±	3	11	±	3
	Female	22	±	10	18	±	12	21	±	14	22	±	11	11	±	4	11	±	5
2-Hydroxyisobutyrate	Male	12	±	2	11	±	2	13	±	2	16	±	3	14	±	2	19	±	5
	Female	3	±	1	1	±	0	3	±	2	5	±	4	2	±	1	4	±	1
2-Oxoglutarate	Male	49	±	9	46	±	9	61	±	22	52	±	21	67	±	11	85	±	11
	Female	44	±	9	48	±	25	48	±	9	41	±	15	55	±	12	60	±	16
2-Oxoisocaproate	Male	6	±	1	7	±	1	5	±	1	8	±	2	6	±	2	5	±	0
	Female	7	±	2	9	±	5	4	±	1	6	±	1	6	±	1	5	±	2
3-Hydroxybutyrate	Male	95	±	90	43	±	12	59	±	29	195	±	233	56	±	21	59	±	31
	Female	170	±	157	87	±	72	89	±	67	176	±	60	121	±	123	91	±	17
3-Hydroxyisobutyrate	Male	44	±	8	43	±	8	35	±	10	41	±	10	32	±	9	20	±	4
	Female	42	±	20	59	±	16	27	±	8	36	±	9	26	±	5	21	±	7
Acetate	Male	48	±	22	62	±	24	59	±	6	45	±	8	53	±	6	56	±	6
	Female	74	±	45	80	±	40	58	±	9	51	±	12	58	±	6	62	±	4
Acetoacetate	Male	15	±	13	11	±	8	6	±	2	26	±	21	10	±	4	6	±	2
	Female	23	±	24	20	±	13	7	±	4	22	±	7	21	±	19	10	±	2
Acetone	Male	6	±	2	5	±	1	3	±	1	10	±	4	6	±	2	2	±	1
	Female	5	±	2	7	±	3	3	±	1	6	±	3	9	±	6	2	±	1
Alanine	Male	490	±	98	617	±	77	680	±	101	495	±	92	550	±	95	727	±	73
	Female	464	±	151	552	±	127	567	±	112	413	±	65	473	±	105	624	±	113
Arginine	Male	131	±	50	218	±	71	158	±	52	188	±	67	106	±	37	88	±	22
	Female	149	±	21	227	±	79	109	±	24	142	±	45	115	±	45	53	±	4
Asparagine	Male	52	±	13	87	±	30	71	±	20	61	±	27	72	±	18	68	±	12
	Female	28	±	28	77	±	29	58	±	22	39	±	29	56	±	10	53	±	17
Aspartate	Male	75	±	13	101	±	43	112	±	14	84	±	35	117	±	35	117	±	19
	Female	35	±	10	55	±	15	84	±	14	46	±	18	74	±	10	72	±	7
Betaine	Male	31	±	6	30	±	8	30	±	2	33	±	6	27	±	3	32	±	5
	Female	34	±	10	32	±	10	54	±	12	43	±	6	36	±	5	45	±	2
Carnitine	Male	26	±	6	44	±	31	33	±	3	29	±	9	30	±	4	38	±	3
	Female	33	±	6	45	±	15	35	±	4	36	±	4	29	±	4	34	±	1
Choline	Male	124	±	14	154	±	27	204	±	32	131	±	17	180	±	22	199	±	33
	Female	122	±	12	140	±	16	191	±	55	138	±	7	164	±	12	149	±	12
Citrate	Male	309	±	29	236	±	79	265	±	26	320	±	56	267	±	24	260	±	47
	Female	210	±	81	241	±	14	283	±	83	284	±	33	292	±	17	281	±	33
Creatine	Male	253	±	60	255	±	36	323	±	52	287	±	66	235	±	32	280	±	42
	Female	263	±	39	259	±	35	317	±	76	225	±	31	274	±	28	280	±	30
Ethanol (contaminant)	Male	16	±	7	48	±	43	31	±	30	352	±	959	16	±	4	19	±	12
	Female	8	±	7	324	±	124	17	±	10	7	±	11	212	±	237	17	±	3
Formate	Male	70	±	12	113	±	42	76	±	12	82	±	21	68	±	11	77	±	17
	Female	62	±	9	63	±	5	70	±	19	90	±	37	66	±	8	93	±	26
Fumarate	Male	15	±	5	20	±	9	16	±	3	11	±	3	11	±	4	12	±	3
	Female	17	±	4	12	±	4	11	±	3	15	±	6	8	±	2	8	±	2
Glucose	Male	7044	±	1474	5714	±	1096	4669	±	770	9470	±	2263	6407	±	1033	5278	±	1231
	Female	5744	±	1273	5660	±	1422	5142	±	1165	8421	±	874	6213	±	851	7725	±	1532
Glutamate	Male	206	±	30	259	±	66	279	±	33	233	±	53	254	±	42	233	±	45
	Female	156	±	33	204	±	54	207	±	38	155	±	14	171	±	15	153	±	13
Glutamine	Male	498	±	67	603	±	98	556	±	58	570	±	58	570	±	48	655	±	45
	Female	645	±	97	648	±	81	620	±	90	681	±	79	612	±	62	586	±	109

Metabolite	Sex	Control Diet									Western Diet								
		5 months			10 months			15 months			5 months			10 months			15 months		
Glycerol	Male	1096	±	172	1010	±	261	786	±	113	1233	±	222	821	±	142	803	±	85
	Female	1552	±	245	1270	±	459	979	±	226	1486	±	252	885	±	67	984	±	110
Glycine	Male	243	±	42	244	±	48	273	±	37	257	±	56	248	±	36	272	±	29
	Female	216	±	33	200	±	21	230	±	34	234	±	14	219	±	29	254	±	44
Glycolate	Male	51	±	12	42	±	5	29	±	7	61	±	20	58	±	11	26	±	7
	Female	33	±	12	40	±	10	26	±	5	59	±	26	59	±	5	34	±	12
Histidine	Male	61	±	16	61	±	30	72	±	24	61	±	19	54	±	27	66	±	15
	Female	74	±	15	48	±	8	67	±	16	60	±	15	50	±	4	54	±	17
Isoleucine	Male	103	±	23	126	±	18	135	±	23	97	±	27	115	±	22	111	±	18
	Female	99	±	10	121	±	24	107	±	11	95	±	20	102	±	13	112	±	23
Lactate	Male	13930	±	1526	13294	±	1929	19203	±	1809	13148	±	1662	12117	±	1540	17148	±	2643
	Female	11140	±	1189	10884	±	1406	16612	±	2222	10651	±	1311	11564	±	590	13258	±	2562
Leucine	Male	160	±	36	217	±	45	193	±	33	163	±	55	204	±	48	172	±	23
	Female	145	±	14	178	±	43	140	±	16	138	±	32	154	±	23	135	±	31
Lysine	Male	513	±	122	579	±	84	631	±	140	415	±	91	445	±	85	446	±	90
	Female	500	±	60	591	±	95	558	±	118	425	±	86	452	±	80	482	±	98
Malate	Male	110	±	43	140	±	48	144	±	27	112	±	37	108	±	28	102	±	21
	Female	107	±	16	135	±	51	103	±	28	66	±	32	81	±	19	71	±	17
Methionine	Male	153	±	51	164	±	23	160	±	39	119	±	29	117	±	33	123	±	40
	Female	137	±	55	185	±	76	123	±	38	98	±	29	113	±	34	124	±	7
N-Methylhydantoin	Male	4	±	1	5	±	2	4	±	0	10	±	6	9	±	1	11	±	2
	Female	7	±	4	8	±	2	7	±	2	14	±	6	10	±	1	12	±	2
O-Acetylcarnitine	Male	14	±	3	13	±	4	15	±	2	15	±	3	13	±	2	15	±	2
	Female	17	±	5	17	±	9	16	±	2	18	±	2	15	±	3	15	±	1
Ornithine	Male	78	±	30	97	±	13	134	±	47	85	±	23	218	±	56	209	±	35
	Female	41	±	10	82	±	17	161	±	56	91	±	10	149	±	56	207	±	45
Phenylalanine	Male	74	±	16	101	±	31	94	±	11	82	±	26	88	±	20	88	±	9
	Female	63	±	18	75	±	14	75	±	16	62	±	10	70	±	8	71	±	11
Proline	Male	179	±	55	245	±	58	190	±	70	184	±	40	193	±	54	141	±	29
	Female	169	±	66	257	±	77	127	±	32	139	±	33	149	±	34	128	±	30
Pyruvate	Male	162	±	60	221	±	67	510	±	67	195	±	73	149	±	50	431	±	48
	Female	138	±	13	180	±	79	433	±	72	183	±	25	251	±	64	417	±	59
Serine	Male	179	±	41	260	±	58	200	±	40	196	±	52	226	±	51	198	±	39
	Female	189	±	83	221	±	61	186	±	30	155	±	45	186	±	25	148	±	36
Succinate	Male	36	±	9	34	±	8	56	±	13	35	±	9	30	±	8	42	±	7
	Female	31	±	5	32	±	12	47	±	16	32	±	9	29	±	4	44	±	11
Taurine	Male	1123	±	109	1095	±	130	1425	±	93	1066	±	173	1136	±	113	1318	±	201
	Female	971	±	138	865	±	68	1228	±	91	878	±	125	1005	±	67	1099	±	112
Threonine	Male	298	±	74	335	±	29	316	±	79	271	±	60	272	±	46	225	±	43
	Female	279	±	69	289	±	100	238	±	65	260	±	50	223	±	27	223	±	38
Trimethylamine	Male	10	±	3	10	±	3	13	±	3	2	±	1	3	±	1	3	±	2
	Female	1	±	0	2	±	2	5	±	3	1	±	1	0	±	0	1	±	0
Trimethylamine-N-oxide	Male	6	±	2	10	±	3	11	±	1	9	±	3	7	±	1	9	±	3
	Female	39	±	5	21	±	10	14	±	3	10	±	6	9	±	1	7	±	0
Tryptophan	Male	48	±	10	51	±	18	47	±	10	57	±	20	59	±	14	42	±	6
	Female	48	±	10	55	±	14	33	±	5	48	±	12	56	±	6	39	±	5
Tyrosine	Male	149	±	48	203	±	47	177	±	41	125	±	37	128	±	35	116	±	19
	Female	128	±	49	139	±	39	120	±	39	91	±	27	106	±	14	104	±	21
Urea	Male	5956	±	936	5241	±	501	5664	±	977	5662	±	1416	4926	±	1235	4785	±	1027
	Female	6587	±	1117	5238	±	591	4530	±	925	4565	±	882	4209	±	371	4631	±	801
Valine	Male	294	±	61	340	±	53	348	±	62	262	±	66	306	±	56	297	±	42
	Female	281	±	54	324	±	87	269	±	41	216	±	45	254	±	35	252	±	36

Supplementary Table 3. Summary of linear regression analysis to evaluate the impact of diet, sex and age on urine metabolites. Metabolites with $p < 0.05$ after FDR correction for at least one factor were included in the table, and p-values for each term are provided.

Metabolite	Diet	Age	Sex	Diet:Age	Diet:Sex	Age:Sex	Diet:Age :Sex
N-Methyl-2-pyridone-5-carboxamide	1.45E-08	3.94E-02	9.29E-01	1.42E-01	1.84E-02	5.95E-05	6.16E-01
N-Methyl-4-pyridone-3-carboxamide	4.25E-09	3.19E-02	9.50E-01	1.26E-01	1.28E-02	7.71E-05	5.62E-01
1-Methylnicotinamide	7.41E-01	2.43E-02	3.12E-16	2.48E-01	1.26E-01	1.08E-05	5.03E-01
2-Hydroxyisobutyrate	1.97E-03	1.56E-02	4.96E-02	1.02E-02	2.52E-03	4.09E-02	5.03E-01
2-Hydroxyvalerate	8.49E-10	9.05E-01	3.60E-02	4.48E-02	1.84E-02	2.42E-02	2.62E-01
2-Oxoisocaproate	9.84E-01	1.64E-03	3.71E-40	3.22E-02	3.96E-03	5.97E-04	9.55E-01
3-Indoxyl sulfate	2.57E-01	1.43E-03	7.42E-04	8.93E-01	3.14E-03	1.31E-04	2.62E-01
3-Methyl-2-oxovalerate	3.14E-01	3.58E-01	2.20E-32	7.72E-02	1.84E-02	7.71E-05	5.26E-01
4-Hydroxyphenylacetate	4.13E-02	2.46E-02	4.77E-09	9.07E-02	1.27E-07	2.17E-01	9.55E-01
4-Hydroxyphenyllactate	4.38E-01	6.11E-01	7.73E-04	1.73E-01	6.71E-06	5.10E-04	5.03E-01
5,6-Dihydrouracil	9.84E-05	1.83E-01	1.39E-02	3.80E-03	1.08E-02	1.36E-01	2.62E-01
Acetamide	4.04E-07	4.76E-01	3.93E-03	9.55E-03	3.10E-02	1.33E-02	5.03E-01
Acetate	2.47E-06	1.01E-02	1.85E-02	7.54E-02	2.98E-01	3.56E-01	5.62E-01
Acetoacetate	1.01E-11	5.01E-01	3.01E-01	8.54E-02	1.84E-02	2.36E-01	4.65E-01
Alanine	2.57E-01	5.93E-02	1.80E-02	6.87E-02	1.93E-01	1.69E-01	6.57E-01
Allantoin	7.13E-07	5.42E-02	6.03E-04	9.88E-03	1.93E-01	1.76E-02	5.03E-01
Arabinose	6.13E-02	1.27E-02	5.55E-01	7.54E-02	2.51E-02	3.81E-03	5.03E-01
Ascorbate	5.46E-04	2.48E-08	4.28E-14	4.15E-01	2.20E-02	2.48E-02	8.14E-01
Betaine	2.55E-01	5.79E-01	7.22E-24	1.50E-02	9.13E-04	1.98E-01	5.03E-01
Carnitine	8.37E-01	1.43E-03	1.29E-11	8.29E-02	9.67E-04	1.20E-01	5.03E-01
Choline	3.80E-03	5.42E-02	7.42E-02	1.02E-01	3.19E-05	3.35E-01	2.80E-01
Citrate	9.84E-01	2.05E-01	1.07E-09	1.23E-07	9.04E-03	5.70E-02	5.03E-01
Creatine	8.68E-03	4.15E-01	1.29E-11	2.78E-02	5.59E-02	1.32E-01	2.62E-01
Creatinine	7.87E-03	4.15E-01	9.05E-01	4.33E-02	1.26E-01	1.57E-02	3.47E-01
Formate	2.69E-02	6.31E-03	4.99E-01	7.80E-01	4.13E-02	1.60E-01	2.62E-01
Fucose	1.30E-02	1.59E-03	6.00E-01	7.03E-02	1.04E-02	1.20E-03	2.80E-01
Fumarate	2.57E-01	1.83E-01	7.85E-02	5.58E-01	1.08E-04	7.77E-01	2.62E-01

Metabolite	Diet	Age	Sex	Diet:Age	Diet:Sex	Age:Sex	Diet:Age: Sex
Glucose	5.66E-01	6.27E-01	6.89E-01	7.03E-02	1.28E-02	1.07E-02	5.03E-01
Glycine	9.84E-01	2.68E-04	5.47E-04	1.27E-01	1.02E-01	5.19E-03	8.14E-01
Guanidoacetate	1.12E-04	2.87E-01	5.66E-03	2.92E-03	5.59E-02	1.73E-03	4.79E-01
Hippurate	2.79E-24	2.56E-01	1.88E-11	3.83E-01	7.87E-01	1.36E-01	5.74E-01
Isobutyrate	5.10E-03	5.42E-02	3.63E-02	4.15E-01	1.28E-01	1.20E-03	5.03E-01
Leucine	7.41E-01	1.74E-02	5.06E-01	1.42E-01	1.53E-03	4.44E-04	5.62E-01
Methylamine	5.16E-03	4.27E-03	3.30E-17	7.54E-02	1.26E-04	8.07E-02	6.57E-01
N,N-Dimethylglycine	3.98E-04	2.48E-08	8.10E-21	3.83E-01	3.19E-05	2.54E-01	5.03E-01
N-Carbamoyl-β-alanine	9.38E-17	7.60E-02	8.75E-15	2.13E-05	4.29E-01	4.50E-01	3.59E-01
N-Phenylacetylglycine	2.05E-04	1.07E-02	9.61E-03	5.38E-01	2.19E-02	1.17E-03	2.80E-01
Niacinamide	2.57E-01	7.23E-02	4.64E-05	3.39E-03	1.81E-01	3.71E-03	2.64E-01
Nicotinamide N-oxide	2.24E-11	1.27E-02	9.50E-01	3.05E-01	6.19E-01	1.39E-06	8.69E-01
Pantothenate	6.12E-01	5.75E-02	1.60E-03	4.28E-01	5.85E-02	1.01E-01	2.62E-01
Pseudouridine	6.51E-07	3.12E-01	1.60E-03	3.39E-03	5.68E-02	3.71E-03	4.79E-01
Succinate	8.90E-03	1.46E-01	1.02E-06	9.88E-03	6.03E-03	3.50E-01	8.53E-01
Sucrose	4.71E-17	9.11E-01	5.06E-01	8.17E-02	1.02E-01	3.38E-01	3.71E-01
Tartrate	8.43E-07	7.61E-06	3.66E-07	1.02E-02	5.25E-01	3.86E-03	6.57E-01
Taurine	5.66E-01	4.86E-01	9.61E-03	1.02E-02	3.57E-03	1.17E-03	2.64E-01
Trimethylamine	9.25E-26	7.37E-03	6.21E-35	6.11E-01	6.71E-06	7.78E-06	5.62E-01
Trimethylamine N-oxide	1.25E-33	1.81E-01	1.90E-37	4.15E-01	1.13E-05	5.97E-04	2.62E-01
Uracil	6.13E-02	1.27E-02	1.03E-03	3.26E-02	3.82E-04	5.85E-03	3.05E-02
Urea	2.03E-01	7.97E-01	6.78E-02	1.50E-02	5.20E-01	6.34E-01	2.62E-01
Valine	5.67E-01	5.71E-01	3.64E-06	3.69E-02	1.24E-04	1.20E-01	5.62E-01
cis-Aconitate	1.39E-01	4.15E-01	5.39E-13	8.53E-04	1.21E-02	6.64E-01	5.26E-01
sn-Glycero-3-phosphocholine	5.68E-01	1.01E-02	1.06E-02	4.33E-02	5.69E-04	4.49E-02	5.62E-01
trans-Aconitate	2.57E-01	1.88E-02	4.75E-01	1.44E-01	9.42E-02	2.54E-01	4.56E-01

Supplementary Table 4. Urine metabolite concentrations (μmol/L) from male and female mice on a CD or a WD. Concentrations are expressed with mean ± SD.

Metabolite	Sex	Control Diet									Western Diet								
		5 months			10 months			15 months			5 months			10 months			15 months		
N-Methyl-2-pyridone-5-carboxamide	Male	246	±	70	340	±	79	277	±	101	329	±	80	499	±	124	321	±	139
	Female	255	±	97	189	±	122	380	±	68	458	±	72	436	±	162	477	±	148
N-Methyl-4-pyridone-3-carboxamide	Male	166	±	46	229	±	54	193	±	68	224	±	56	344	±	91	224	±	97
	Female	169	±	66	124	±	80	253	±	46	315	±	50	307	±	121	321	±	98
1-Methylnicotinamide	Male	323	±	133	539	±	144	355	±	135	326	±	114	489	±	154	309	±	170
	Female	744	±	289	524	±	292	1134	±	224	930	±	179	746	±	258	778	±	90
2-Hydroxyisobutyrate	Male	46	±	12	42	±	12	39	±	7	40	±	18	64	±	21	48	±	12
	Female	29	±	12	31	±	22	47	±	7	46	±	19	70	±	35	67	±	32
2-Hydroxyvalerate	Male	135	±	57	137	±	49	102	±	45	187	±	59	225	±	77	169	±	62
	Female	118	±	66	72	±	89	102	±	35	151	±	59	301	±	219	304	±	147
2-Oxoisocaproate	Male	304	±	98	177	±	99	181	±	90	255	±	105	186	±	101	95	±	23
	Female	973	±	366	868	±	541	1207	±	210	1196	±	207	1688	±	954	1141	±	148
3-Indoxyl sulfate	Male	752	±	286	1270	±	290	1186	±	835	579	±	161	868	±	209	1051	±	308
	Female	1172	±	522	745	±	560	1301	±	186	1222	±	362	1216	±	345	1295	±	407
3-Methyl-2-oxovalerate	Male	227	±	53	171	±	45	147	±	49	204	±	47	189	±	54	126	±	41
	Female	532	±	215	440	±	308	707	±	125	553	±	204	896	±	433	870	±	201
4-Hydroxyphenylacetate	Male	239	±	57	210	±	106	181	±	62	158	±	50	127	±	66	74	±	35
	Female	81	±	41	87	±	60	81	±	21	159	±	65	146	±	45	87	±	6
4-Hydroxyphenyllactate	Male	110	±	38	86	±	32	77	±	15	69	±	20	77	±	24	56	±	25
	Female	50	±	18	55	±	39	87	±	13	81	±	14	75	±	19	97	±	7
5,6-Dihydrouracil	Male	196	±	61	209	±	63	176	±	34	199	±	57	306	±	98	215	±	100
	Female	240	±	124	150	±	108	276	±	79	302	±	127	548	±	257	288	±	87
Acetamide	Male	184	±	65	170	±	40	163	±	37	202	±	59	300	±	77	219	±	62
	Female	242	±	123	153	±	150	274	±	53	308	±	124	478	±	332	476	±	240
Acetate	Male	130	±	35	140	±	31	147	±	27	156	±	59	245	±	80	198	±	78
	Female	149	±	62	153	±	101	241	±	133	222	±	72	302	±	152	229	±	65
Acetoacetate	Male	112	±	47	106	±	29	91	±	41	162	±	54	196	±	60	132	±	38
	Female	101	±	55	71	±	65	84	±	24	159	±	76	270	±	168	239	±	132
Alanine	Male	116	±	32	119	±	39	159	±	130	111	±	38	151	±	44	122	±	52
	Female	139	±	74	122	±	77	208	±	48	161	±	39	167	±	41	157	±	30
Allantoin	Male	13698	±	4171	15919	±	4556	13997	±	3255	15707	±	4352	28751	±	7242	21827	±	6998
	Female	23159	±	10912	14062	±	12885	22984	±	3789	25953	±	11907	39857	±	19455	37134	±	16575
Arabinose	Male	319	±	103	409	±	137	309	±	100	295	±	96	469	±	113	340	±	165
	Female	329	±	134	257	±	187	444	±	102	380	±	169	535	±	212	537	±	201
Ascorbate	Male	1624	±	711	1072	±	836	657	±	457	2062	±	1260	1840	±	1622	878	±	616
	Female	409	±	302	345	±	327	28	±	11	1058	±	773	1392	±	1716	192	±	207
Betaine	Male	176	±	39	127	±	51	140	±	45	135	±	32	148	±	45	128	±	44
	Female	322	±	162	267	±	144	367	±	77	413	±	98	615	±	363	353	±	30
Carnitine	Male	74	±	28	64	±	23	96	±	53	47	±	16	72	±	34	64	±	23
	Female	25	±	14	40	±	32	53	±	13	36	±	9	55	±	15	46	±	2
Choline	Male	85	±	25	76	±	26	59	±	13	80	±	12	81	±	31	59	±	23
	Female	73	±	30	57	±	28	78	±	21	156	±	123	150	±	56	78	±	21
Citrate	Male	436	±	271	1052	±	3350	3888	±	6018	521	±	476	370	±	361	103	±	30
	Female	837	±	946	1892	±	2186	2480	±	1344	5635	±	7160	3227	±	3220	471	±	525
Creatine	Male	613	±	444	693	±	462	963	±	936	843	±	368	1524	±	2069	238	±	137
	Female	1680	±	1160	1239	±	902	1853	±	683	2528	±	1644	4300	±	3301	3596	±	980
Creatinine	Male	3392	±	967	3591	±	876	2759	±	667	3295	±	818	4629	±	1242	3438	±	1151
	Female	3589	±	1443	2479	±	1851	3798	±	678	3945	±	1590	4873	±	1544	3946	±	1536
Formate	Male	130	±	58	280	±	343	89	±	40	78	±	20	123	±	59	103	±	58
	Female	113	±	75	144	±	86	157	±	61	133	±	53	154	±	59	111	±	75
Fucose	Male	407	±	126	583	±	137	395	±	111	386	±	113	676	±	185	475	±	173
	Female	447	±	203	334	±	279	548	±	107	491	±	242	809	±	329	936	±	498

Metabolite	Sex	Control Diet									Western Diet								
		5 months			10 months			15 months			5 months			10 months			15 months		
Fumarate	Male	13	±	3	17	±	18	14	±	10	6	±	2	11	±	5	10	±	2
	Female	12	±	7	15	±	13	13	±	6	33	±	50	22	±	18	13	±	3
Glucose	Male	1613	±	595	1380	±	432	1180	±	268	1071	±	346	1440	±	474	1016	±	370
	Female	1270	±	632	1162	±	847	1966	±	477	1423	±	407	1712	±	652	1553	±	391
Glycine	Male	304	±	106	258	±	134	233	±	235	249	±	109	308	±	171	119	±	50
	Female	219	±	87	89	±	27	150	±	71	282	±	68	280	±	479	109	±	30
Guanidoacetate	Male	565	±	189	388	±	124	317	±	92	520	±	159	694	±	268	389	±	131
	Female	526	±	210	381	±	252	702	±	186	739	±	248	893	±	297	694	±	317
Hippurate	Male	317	±	92	345	±	86	306	±	100	681	±	165	1030	±	303	834	±	238
	Female	611	±	297	551	±	384	693	±	251	1428	±	309	1423	±	491	1330	±	211
Isobutyrate	Male	36	±	13	41	±	13	24	±	13	39	±	13	55	±	18	32	±	12
	Female	36	±	22	41	±	39	56	±	14	45	±	24	99	±	39	77	±	21
Leucine	Male	73	±	19	49	±	12	44	±	25	61	±	20	50	±	19	30	±	11
	Female	55	±	42	39	±	22	52	±	9	52	±	11	74	±	22	81	±	24
Methylamine	Male	353	±	115	297	±	112	230	±	129	299	±	77	353	±	134	225	±	73
	Female	151	±	59	86	±	53	110	±	34	226	±	70	219	±	80	166	±	38
N,N-Dimethylglycine	Male	27	±	11	13	±	9	10	±	5	25	±	9	16	±	9	11	±	7
	Female	50	±	29	30	±	16	28	±	6	127	±	38	142	±	120	44	±	10
N-Carbamoyl-β-alanine	Male	1114	±	575	972	±	295	1237	±	613	1640	±	569	3063	±	864	2238	±	940
	Female	2522	±	1317	1476	±	1134	2721	±	789	3459	±	957	7940	±	4370	3904	±	1325
N-Phenylacetylglycine	Male	933	±	257	1519	±	463	1211	±	702	1079	±	305	1741	±	656	1624	±	683
	Female	1418	±	688	934	±	756	1507	±	294	1841	±	526	2195	±	933	2199	±	507
Niacinamide	Male	125	±	54	154	±	38	114	±	50	114	±	31	131	±	43	63	±	34
	Female	156	±	51	166	±	70	210	±	50	276	±	46	136	±	90	116	±	60
Nicotinamide N-oxide	Male	243	±	116	425	±	123	319	±	139	453	±	189	818	±	193	462	±	225
	Female	303	±	120	216	±	148	448	±	110	684	±	182	430	±	247	658	±	152
Pantothenate	Male	360	±	107	339	±	101	338	±	88	258	±	83	369	±	125	285	±	97
	Female	199	±	96	309	±	222	377	±	68	318	±	122	282	±	190	286	±	121
Pseudouridine	Male	255	±	77	270	±	75	217	±	51	264	±	63	441	±	114	320	±	104
	Female	354	±	153	211	±	173	339	±	68	424	±	174	556	±	218	511	±	211
Succinate	Male	95	±	38	150	±	196	267	±	277	119	±	42	148	±	68	99	±	31
	Female	144	±	66	183	±	135	196	±	56	347	±	245	432	±	99	165	±	20
Sucrose	Male	1004	±	605	759	±	241	846	±	188	2210	±	558	2863	±	1545	2011	±	1304
	Female	646	±	366	971	±	861	1127	±	320	3147	±	1362	3441	±	3061	1856	±	1176
Tartrate	Male	61	±	19	86	±	24	63	±	39	69	±	26	162	±	50	109	±	39
	Female	40	±	17	39	±	27	57	±	13	47	±	19	86	±	24	97	±	33
Taurine	Male	20743	±	9142	21846	±	6343	20365	±	6266	14237	±	4245	27112	±	11644	18573	±	8436
	Female	16798	±	7525	9747	±	7219	17059	±	6143	24976	±	6914	20336	±	7141	10466	±	4104
Trimethylamine	Male	13119	±	3300	13823	±	2701	10554	±	3862	2786	±	788	2990	±	1348	3028	±	1326
	Female	1706	±	1342	1730	±	1274	4212	±	1378	724	±	329	1267	±	908	2827	±	2291
Trimethylamine N-oxide	Male	1370	±	408	1812	±	286	1764	±	399	519	±	138	768	±	212	762	±	292
	Female	8359	±	3163	7739	±	4473	8548	±	1581	2204	±	795	1222	±	602	1114	±	754
Uracil	Male	76	±	22	69	±	19	41	±	13	66	±	20	70	±	26	57	±	52
	Female	92	±	43	35	±	18	79	±	21	106	±	49	154	±	76	93	±	41
Urea	Male	392082	±	131041	300397	±	73851	375438	±	81369	360902	±	64393	423164	±	124950	308331	±	80170
	Female	368292	±	143589	408250	±	193902	499097	±	127635	519753	±	152073	440133	±	198278	325254	±	32316
Valine	Male	53	±	13	44	±	9	58	±	73	43	±	13	45	±	11	30	±	9
	Female	56	±	31	48	±	22	67	±	14	75	±	15	114	±	49	63	±	6
cis-Aconitate	Male	841	±	238	862	±	742	1173	±	466	814	±	236	1095	±	454	594	±	131
	Female	1257	±	524	1671	±	1176	2128	±	402	2422	±	918	2767	±	812	1242	±	249
sn-Glycero-3-phosphocholine	Male	118	±	31	122	±	36	139	±	103	87	±	27	134	±	58	103	±	34
	Female	75	±	35	69	±	48	127	±	32	92	±	41	176	±	98	153	±	44
trans-Aconitate	Male	120	±	46	111	±	38	154	±	71	118	±	88	141	±	49	136	±	56
	Female	122	±	99	119	±	120	282	±	202	179	±	84	167	±	83	149	±	39

Supplementary Table 5. Summary of multivariate response linear regression analysis on the balance of microbial communities. The effect of covariates, diet and sex, on the microbiome of fecal and cecal samples collected at 5, 10 and 15 months were evaluated separately using Gneiss. To estimate the effect of a single covariate, a leave-one-variable-out approach was used and the results reported as R^2diff.

	Age	Diet (R^2diff)	Sex (R^2diff)	R^2
**Fecal**	5	0.0899	0.204	0.294
	10	0.0863	0.147	0.233
	15	0.0747	0.254	0.335
**Cecal**	5	0.110	0.178	0.288
	10	0.0851	0.144	0.229
	15	0.119	0.246	0.379

Supplementary Table 6. Fecal microbes significantly different in abundance between male and female mice accounting for diet. Differential abundance analysis was performed using gneiss, in combination with OLS regression models to identify taxa significantly different between groups.

	5 months	10 months	15 months
**Phylum**	*Actinobacteria*	*Actinobacteria*	*Actinobacteria*
	Bacteroidetes	*Bacteroidetes*	*Bacteroidetes*
	Firmicutes	*Firmicutes*	*Firmicutes*
	Proteobacteria	*Proteobacteria*	*Proteobacteria*
	Tenericutes		
	Verrucomicrobia		
**Family**	*Alcaligenaceae*	*S24-7*	*S24-7*
	Bacteroidaceae	*Bacteroidaceae*	*Bifidobacteriaceae*
	Bifidobacteriaceae	*Clostridiaceae*	*Clostridiaceae*
	Christensenellaceae	*Coriobacteriaceae*	*Coriobacteriaceae*
	Clostridiaceae	*Desulfovibrionaceae*	*Dehalobacteriaceae*
	Coriobacteriaceae	*Erysipelotrichaceae*	*Desulfovibrionaceae*
	Dehalobacteriaceae	*Helicobacteraceae*	*Erysipelotrichaceae*
	Erysipelotrichaceae	*Lachnospiraceae*	*Helicobacteraceae*
	S24-7	*Lactobacillaceae*	*Lachnospiraceae*
	Lachnospiraceae	*Rikenellaceae*	*Lactobacillaceae*
	Lactobacillaceae	*Ruminococcaceae*	*Mogibacteriaceae*
	Mogibacteriaceae	*Streptococcaceae*	*Porphyromonadaceae*
	Peptostreptococcaceae	*Turicibacteraceae*	*Rikenellaceae*
	Rikenellaceae	*Verrucomicrobiaceae*	*Ruminococcaceae*
	Ruminococcaceae		*Verrucomicrobiaceae*
	Verrucomicrobiaceae		
**Genus**	*Adlercreutzia*	*Akkermansia*	*AF12*
	AF12	*Bacteroides*	*Akkermansia*
	Akkermansia	*Bilophila*	*Allobaculum*
	Allobaculum	*Coprococcus*	*Bifidobacterium*
	Anaerotruncus	*Helicobacter*	*Coprococcus*
	Bacteroides	*Lactobacillus*	*Dehalobacterium*
	Bifidobacterium	*Lactococcus*	*Helicobacter*
	Blautia	*Oscillospira*	*Lactobacillus*
	Coprococcus	*Ruminococcus*	*Oscillospira*
	Dehalobacterium	*Turicibacter*	*Parabacteroides*
	Dorea		*Roseburia*
	Eubacterium		*Ruminococcus*
	Lactobacillus		
	Oscillospira		
	Ruminococcus		
	Sutterella		

Supplementary Table 7. Fecal microbes significantly in abundance between CD and WD accounting for sex. Differential abundance analysis was performed using gneiss, in combination with OLS regression models to identify taxa significantly different between groups.

	5 months	10 months	15 months
**Phylum**	*Actinobacteria*	*Actinobacteria*	*Bacteroidetes*
	Bacteroidetes	*Bacteroidetes*	*Firmicutes*
	Firmicutes	*Firmicutes*	*Verrucomicrobia*
	Proteobacteria	*Tenericutes*	
	Tenericutes		
	Verrucomicrobia		
**Family**	*Alcaligenaceae*	*Clostridiaceae*	*Erysipelotrichaceae*
	Anaeroplasmataceae	*Coriobacteriaceae*	*Lachnospiraceae*
	Bacteroidaceae	*Erysipelotrichaceae*	*Mogibacteriaceae*
	Bifidobacteriaceae	*Lachnospiraceae*	*Peptococcaceae*
	Christensenellaceae	*Peptococcaceae*	*Porphyromonadaceae*
	Clostridiaceae	*Ruminococcaceae*	*Ruminococcaceae*
	Coriobacteriaceae	*Ruminococcaceae*	*S24-7*
	Desulfovibrionaceae	*S24-7*	*Verrucomicrobiaceae*
	Enterococcaceae	*Staphylococcaceae*	
	Erysipelotrichaceae	*Streptococcaceae*	
	f-S24-7	*Turicibacteraceae*	
	Lachnospiraceae		
	Lactobacillaceae		
	Mogibacteriaceae		
	Peptostreptococcaceae		
	Rikenellaceae		
	Ruminococcaceae		
	Streptococcaceae		
	Verrucomicrobiaceae		
**Genus**	*Akkermansia*	*Sutterella*	*Akkermansia*
	Allobaculum	*Adlercreutzia*	*Oscillospira*
	Anaeroplasma	*Allobaculum*	*Parabacteroides*
	Anaerotruncus	*Coprococcus*	*Roseburia*
	Bacteroides	*Lactococcus*	
	Bifidobacterium	*Oscillospira*	
	Clostridium	*Roseburia*	
	Coprobacillus	*Ruminococcus*	
	Coprococcus	*Staphylococcus*	
	Enterococcus	*Turicibacter*	
	Eubacterium		
	Lactobacillus		
	Lactococcus		
	Oscillospira		
	Ruminococcus		
	Sutterella		

Supplementary Table 8. Cecal microbes identified as significantly different in abundance between male and female mice accounting for diet. Differential abundance analysis was performed using gneiss, in combination with ordinary least squares (OLS) regression models to identify taxa significantly different between groups.

	5 months	10 months	15 months
**Phylum**	*Bacteroidetes*	*Actinobacteria*	*Actinobacteria*
	Firmicutes	*Bacteroidetes*	*Bacteroidetes*
	Proteobacteria	*Firmicutes*	*Deferribacteres*
	Tenericutes	*Proteobacteria*	*Firmicutes*
		Tenericutes	*Proteobacteria*
			Tenericutes
			Verrucomicrobia
**Family**	*Alcaligenaceae*	*Christensenellaceae*	*S24-7*
	Bacteroidaceae	*Clostridiaceae*	*Anaeroplasmataceae*
	Christensenellaceae	*Coriobacteriaceae*	*Bacteroidaceae*
	Clostridiaceae	*Dehalobacteriaceae*	*Bifidobacteriaceae*
	Dehalobacteriaceae	*Desulfovibrionaceae*	*Christensenellaceae*
	Desulfovibrionaceae	*Erysipelotrichaceae*	*Clostridiaceae*
	Enterococcaceae	*Helicobacteraceae*	*Coriobacteriaceae*
	Erysipelotrichaceae	*Lachnospiraceae*	*Deferribacteraceae*
	Lachnospiraceae	*Lactobacillaceae*	*Desulfovibrionaceae*
	Lactobacillaceae	*Mogibacteriaceae*	*Erysipelotrichaceae*
	Peptostreptococcaceae	*Rikenellaceae*	*Lachnospiraceae*
	Rikenellaceae	*Ruminococcaceae*	*Lactobacillaceae*
	Ruminococcaceae	*S24-7*	*Peptostreptococcaceae*
	S24-7	*Streptococcaceae*	*Rikenellaceae*
			Ruminococcaceae
			Streptococcaceae
			Turicibacteraceae
			Verrucomicrobiaceae
**Genus**	*Bacteroides*	*Bilophila*	*Akkermansia*
	Coprococcus	*Coprococcus*	*Allobaculum*
	Dehalobacterium	*Dehalobacterium*	*Anaeroplasma*
	Desulfovibrio	*Dorea*	*Anaerotruncus*
	Dorea	*Helicobacter*	*Bacteroides*
	Enterococcus	*Lactobacillus*	*Bifidobacterium*
	Lactobacillus	*Lactococcus*	*Coprococcus*
	Oscillospira	*Oscillospira*	*Desulfovibrio*
	Ruminococcus	*Roseburia*	*Dorea*
	Sutterella	*Ruminococcus*	*Lactobacillus*
			Lactococcus
			Mucispirillum
			Oscillospira
			Roseburia
			Ruminococcus
			Turicibacter

Supplementary Table 9. Cecal microbes significantly in abundance between CD and WD accounting for sex. Differential abundance analysis was performed using gneiss, in combination with OLS regression models to identify taxa significantly different between groups.

	5 months	10 months	15 months
**Phylum**	*Bacteroidetes*	*Bacteroidetes*	*Actinobacteria*
	Firmicutes	*Firmicutes*	*Bacteroidetes*
	Tenericutes	*Proteobacteria*	*Deferribacteres*
		Tenericutes	*Firmicutes*
			Proteobacteria
**Family**	*Bacteroidaceae*	*Clostridiaceae*	*Anaeroplasmataceae*
	Christensenellaceae	*Desulfovibrionaceae*	*Bacteroidaceae*
	Dehalobacteriaceae	*Erysipelotrichaceae*	*Bifidobacteriaceae*
	Erysipelotrichaceae	*Lachnospiraceae*	*Christensenellaceae*
	Lachnospiraceae	*Lactobacillaceae*	*Clostridiaceae*
	Mogibacteriaceae	*Mogibacteriaceae*	*Deferribacteraceae*
	Peptostreptococcaceae	*Rikenellaceae*	*Desulfovibrionaceae*
	Ruminococcaceae	*Ruminococcaceae*	*Helicobacteraceae*
	S24-7	*S24-7*	*Lachnospiraceae*
	Streptococcaceae	*Streptococcaceae*	*Lactobacillaceae*
			Odoribacteraceae
			Peptostreptococcaceae
			Porphyromonadaceae
			Rikenellaceae
			Ruminococcaceae
			S24-7
			Streptococcaceae
			Turicibacteraceae
**Genus**	*Allobaculum*	*Allobaculum*	*Allobaculum*
	Bacteroides	*Bilophila*	*Anaeroplasma*
	Coprococcus	*Coprococcus*	*Anaerotruncus*
	Dehalobacterium	*Dorea*	*Bacteroides*
	Dorea	*Lactobacillus*	*Bifidobacterium*
	Oscillospira	*Lactococcus*	*Coprococcus*
	Ruminococcus	*Oscillospira*	*Desulfovibrio*
	Streptococcus	*Roseburia*	*Dorea*
		Ruminococcus	*Helicobacter*
			Lactobacillus
			Lactococcus
			Mucispirillum
			Odoribacter
			Oscillospira
			Parabacteroides
			Ruminococcus
			Turicibacter

Supplementary Table 10. The nutritional composition of the diets used in this study. The control diet provides 3.6 kcal/g and the Western diet 4.5 kcal/g. The information was obtained from the ENVIGO Teklad Diets website (https://www.envigo.com).

Component	CD		WD	
	% by weight	% kcal from	% by weight	% kcal from
**Protein**	17.3	19.1	17.3	15.2
**Carbohydrate**	61.3	67.9	48.5	42.7
**Fat**	5.2	13	21.2	42

Supplementary Table 11. Formula of the diets used in this study. The control diet (TD.08485) is different from Western diet (TD.88137) with the lower fat and sucrose, and no cholesterol. The information was obtained from the ENVIGO Teklad Diets website (https://www.envigo.com).

Ingredient	CD (g/kg)	WD (g/kg)
Casein	195	195
DL-Methionine	3	3
Sucrose	120	341.46
Corn Starch	432.99	150
Maltodextrin	100	0
Anhydrous Milkfat	37.20	210
Soybean Oil	12.80	0
Cholesterol	0	1.50
Cellulose	50	50
Mineral Mix, AIN-76 (170915)	35	35
Calcium Carbonate	4	4
Vitamin Mix, Teklad (40060)	10	10
Ethoxyquin, antioxidant	0.010	0.040

Supplementary Table 12. The fatty acid (FA) composition of the diets used in this study. 3.7% of total fat comes from milk fat and 1.3% from soybean oil in CD, whereas milk fat is the only fat source in WD. The information was obtained from the ENVIGO Teklad Diets website (https://www.envigo.com) and USDA National Nutrient Databases.

Category	FA	CD (g/kg product)	WD (g/kg product)
**Saturated FAs**	Palmitic acid (C16:0)	11.2<br>[9.86 (milk fat) + 1.34 (soy oil)]	55.7 (milk fat)
	Stearic acid (C18:0)	4.33<br>[3.76 (milk fat) + 0.57 (soy oil)]	21.2 (milk fat)
**Monounsaturated FAs**	Oleic acid (C18:1)	9.57<br>[6.68 (milk fat) + 2.89 (soy oil)]	37.7 (milk fat)
**Polyunsaturated FAs**	Linoleic acid (C18:2)	6.81<br>[0.36 (milk fat) + 6.45 (soy oil)]	2.01 (milk fat)
	Linolenic acid (C18:3)	1<br>[0.13 (milk fat) + 0.87 (soy oil)]	0.72 (milk fat)
**Trans FAs**	NA	1.75<br>[1.75 (milk fat) + 0 (soy oil)]	9.87 (milk fat)
**Cholesterol**	NA	0.089<br>[0.089 (milk fat) + 0 (soy oil)]	2<br>[0.50 (milk fat) + 1.5 (added)]

Supplementary Table 13. Sample sizes for serum, urine, cecal and fecal samples.

		Male[1]					Female[1]			
**Age[1]**	**Diet[1]**	**Feces[2]**	**Urine[3]**	**Serum[4]**	**Cecal contents[5]**		**Feces[2]**	**Urine[3]**	**Serum[4]**	**Cecal contents[5]**
**5**	**Control**	20	19	14	16		20	19	4	6
	**Western**	18	18	13	16		19	7	6	6
**10**	**Control**	15	8	9	16		16	6	8	6
	**Western**	16	15	12	16		16	6	5	6
**15**	**Control**	8	6	8	8		7	8	6	8
	**Western**	8	6	8	8		3	3	3	3

[1]Total number of mice per group was 20, for a total of 120 mice at the beginning of the experiment. Over time, several of the male and female mice (particularly female mice on the WD) developed ulcerative dermatitis, and for humane reasons needed to be euthanized and removed from the study.

[2]Fecal samples were collected after handling of animals immediately prior to necropsy. The number of samples reflects the number of animals at the specific timepoint and diet that produced a sample.

[3]Urine samples were collected after handling of animals immediately prior to necropsy through bladder massage. The number of samples reflects the number of animals at the specific timepoint and diet that produced a sample of enough volume to analyze.

[4]Serum samples were collected at necropsy through cardiac puncture. In some cases, not enough serum was collected to perform metabolomics analysis.

[5]Cecal content was collected at necropsy. In some cases there was not enough cecal content to perform 16S sequencing.

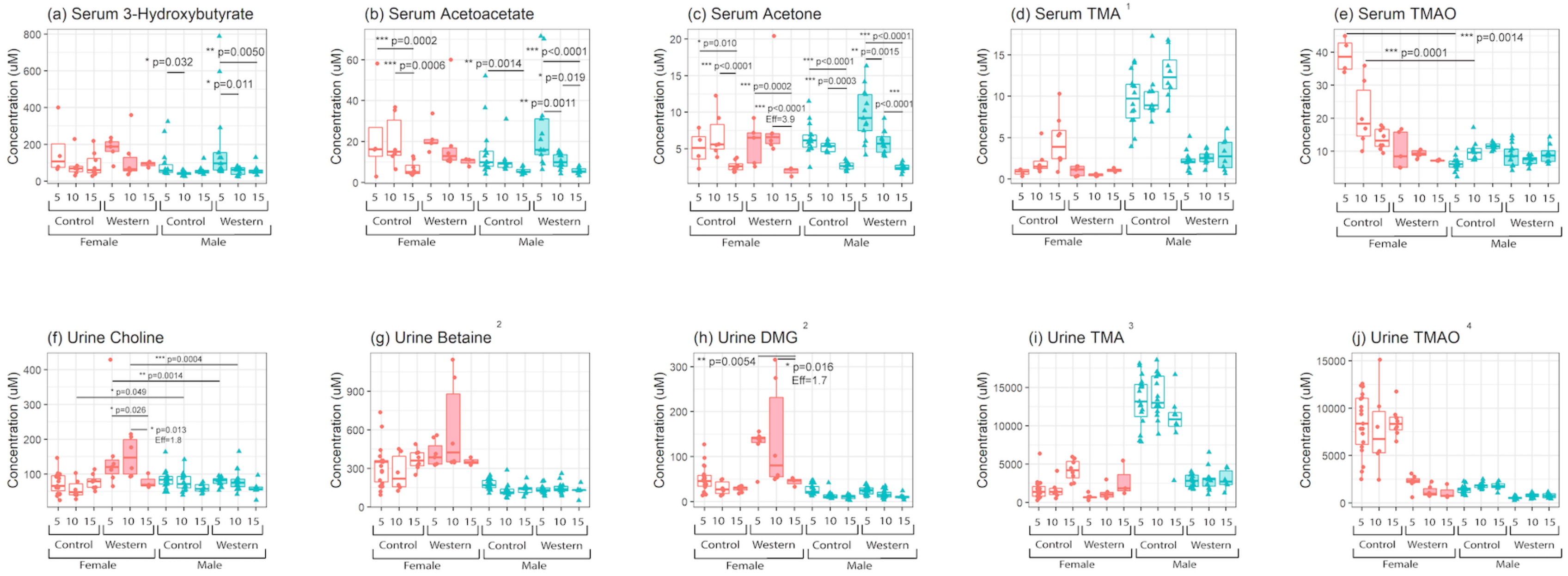

Supplementary Figure 1. Boxplots showing concentrations of either serum or urine metabolites. After fitting a linear model, estimated marginal means (EMMs) were obtained and compared as a post-hoc test ($p<0.05$ were included in the plots). Effect size for female samples at 15 months of age are included. The middle line in the box plot represents the median. Female samples are shown as red circles and male as blue triangles. Mice on the CD are represented with empty boxes and WD with filled boxes. (a) Serum 3-Hydroxybutyrate; (b) serum acetoacetate; (c) serum acetone; (d) serum TMA; (e) serum TMAO; (f) urinary choline; (g) urinary betaine; (h) urinary DMG; (i) urinary TMA; (j) urinary TMAO. *: $p<0.05$; **: $p<0.01$; ***:$p<0.001$.

[1] Male mice showed significantly higher concentration than female mice regardless of age and diet.

[2] Female mice showed significantly higher concentration than male mice regardless of age and diet.

[3] Male mice showed significantly higher concentration than female mice regardless of age and diet, except for mice on the WD at 15 months of age ($p>0.05$).

[4] Female mice showed significantly higher concentration than male mice regardless of age and diet, except for mice on the WD at 15 months of age ($p>0.05$).

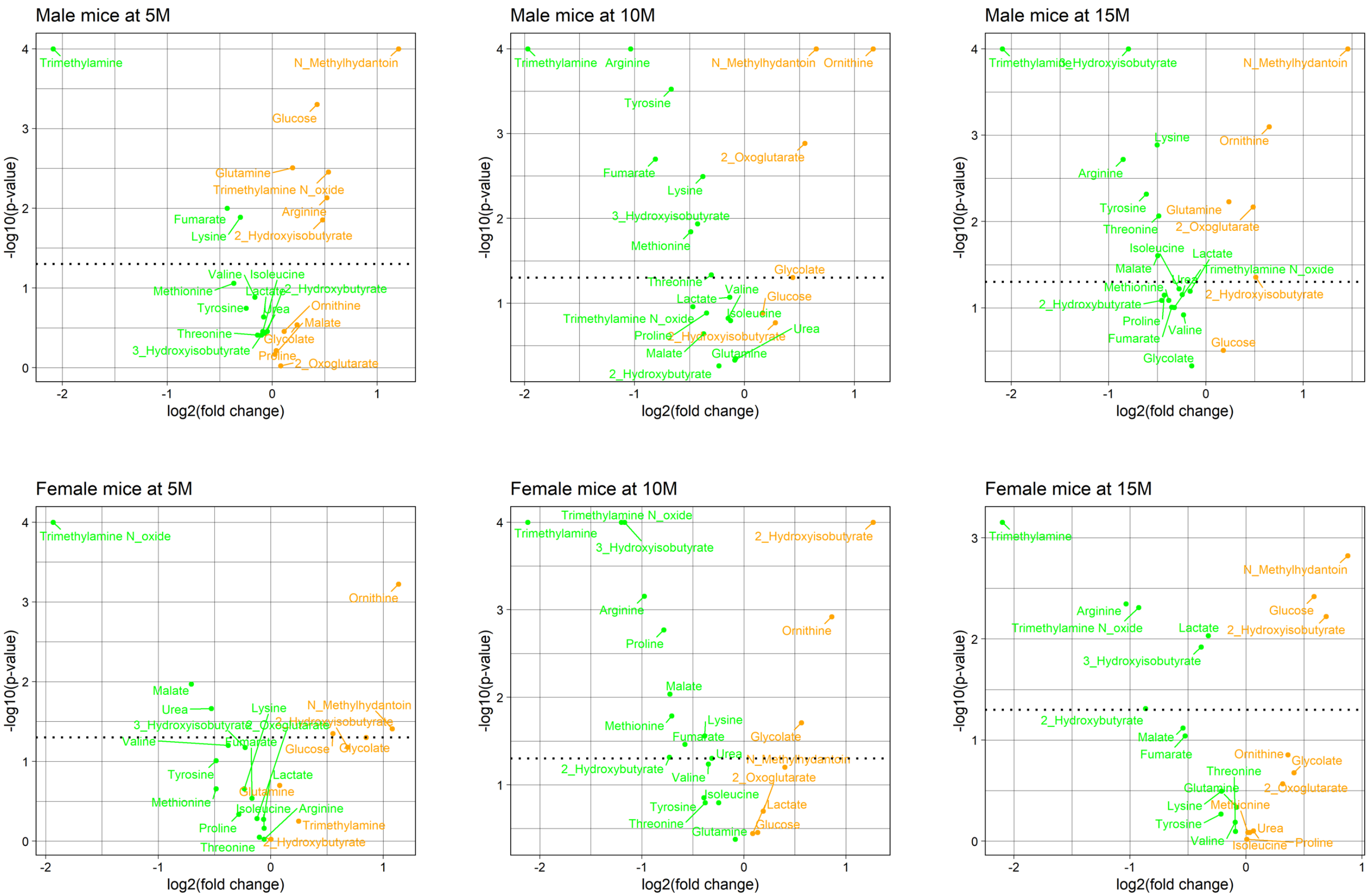

Supplementary Figure 2. Volcano plots showing the difference in the serum metabolome between CD and WD for male and female mice. Among 23 serum metabolites that showed significant impact of diet in Supplementary Table 1, a post-hoc test was applied to test the impact of diet at each age for male and female mice separately. Metabolites with higher levels in the WD group are indicated in orange, whereas those with higher levels in the CD group are indicated in green. Metabolites above the dotted line correspond to those with a p-value < 0.05.

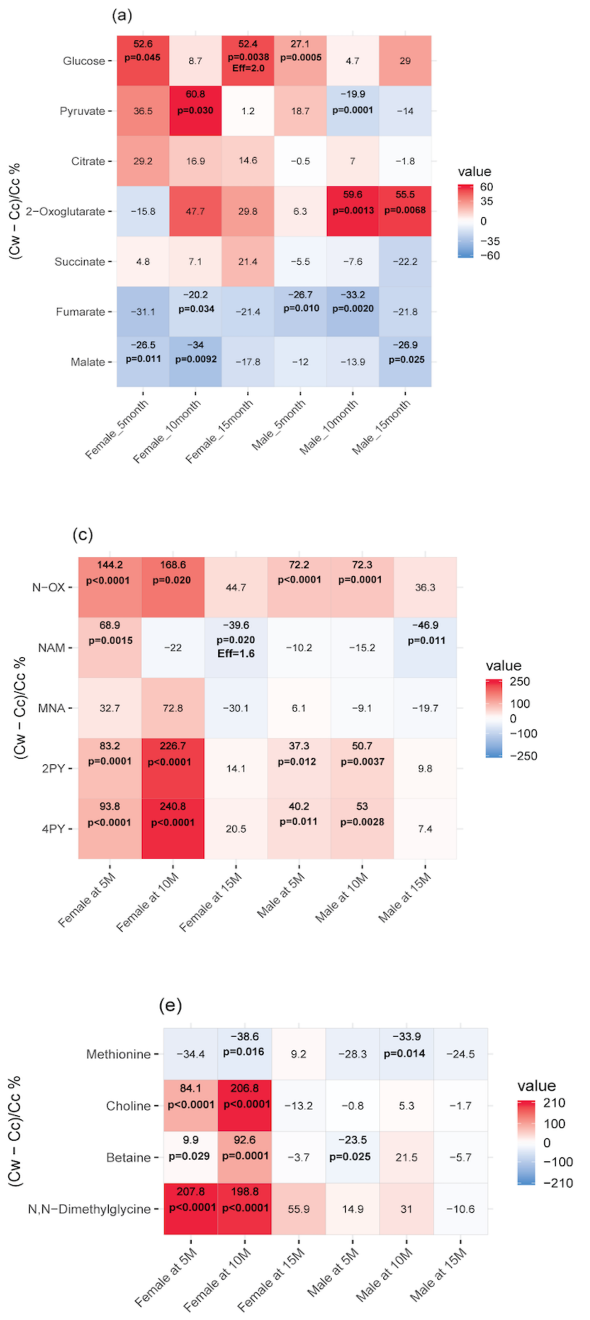

Supplementary Figure 3. Heatmaps showing the rate of increase in the median concentration of metabolites from the WD group (Cw) compared to the CD group (Cc). After fitting a linear model, estimated marginal means (EMMs) were obtained and compared as a post-hoc test ($p<0.05$ were included in the plots before FDR correction). Effect size for female samples at 15 months of age are included. (a) Serum metabolites involved in glycolysis and TCA cycle; (b) serum and urine TMA and TMAO; (c) urinary metabolites involved in NNMT pathway; (d) urinary glucose and TCA cycle intermediates; (e) urinary metabolites associated with the PEMT and BHMT pathways and serum methionine.

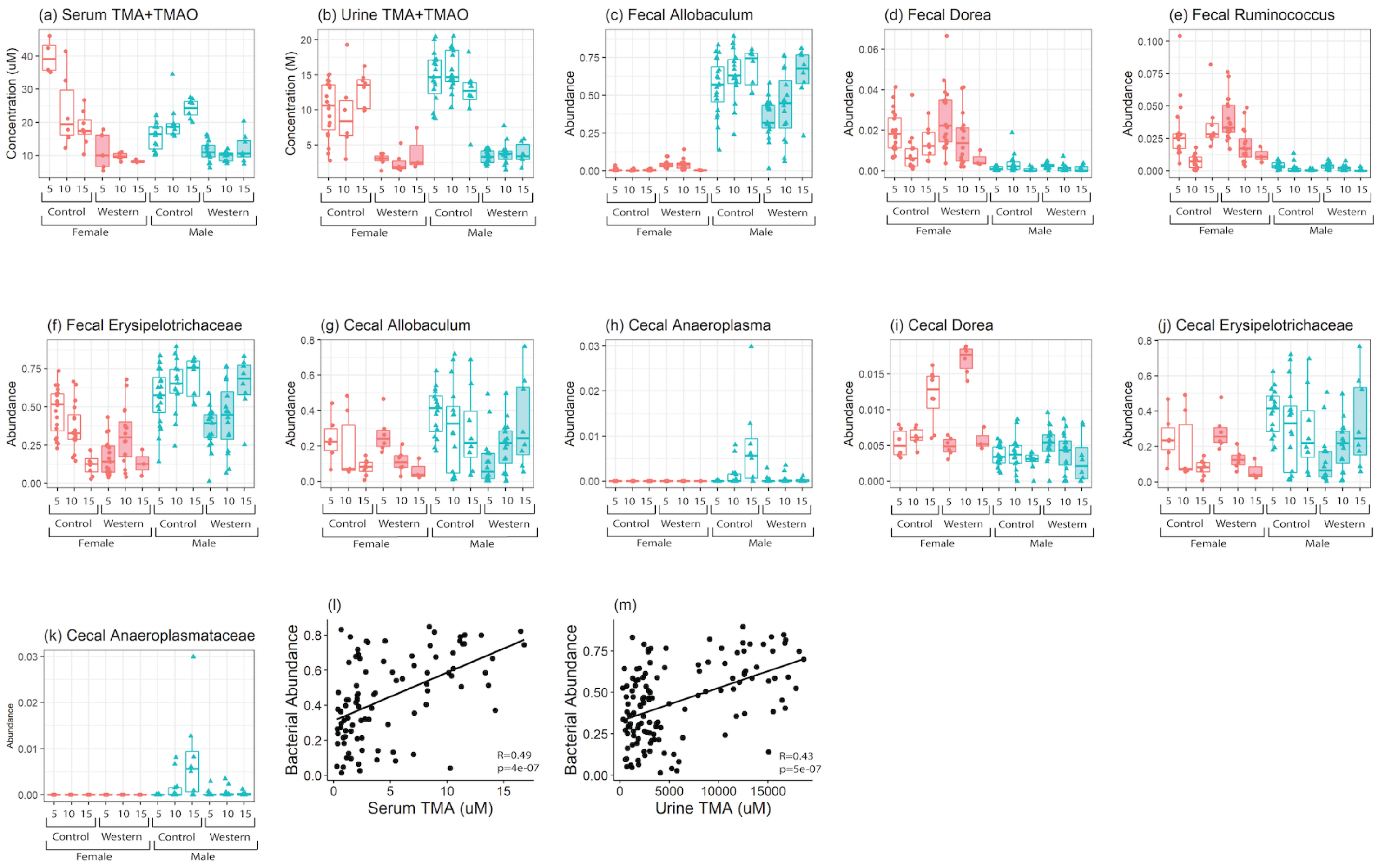

Supplementary Figure 4. Boxplots showing the concentrations of TMA+TMAO from (a) serum or (b) urine samples. Boxplots showing relative abundance of (c) fecal *Allobaculum*; (d) fecal *Dorea*; (e) fecal *Ruminococcus*; (f) fecal *Erysipelotrichaceae*; (g) cecal *Allobaculum*; (h) cecal *Anaeroplasma*; (i) cecal *Dorea*; (j) cecal *Erysipelotrichaceae*; (k) cecal Anaeroplasmataceae. Scatter plots showing the correlation between the relative abundance of fecal *Erysipelotrichaceae* and (l) serum TMA or (m) urinary TMA.

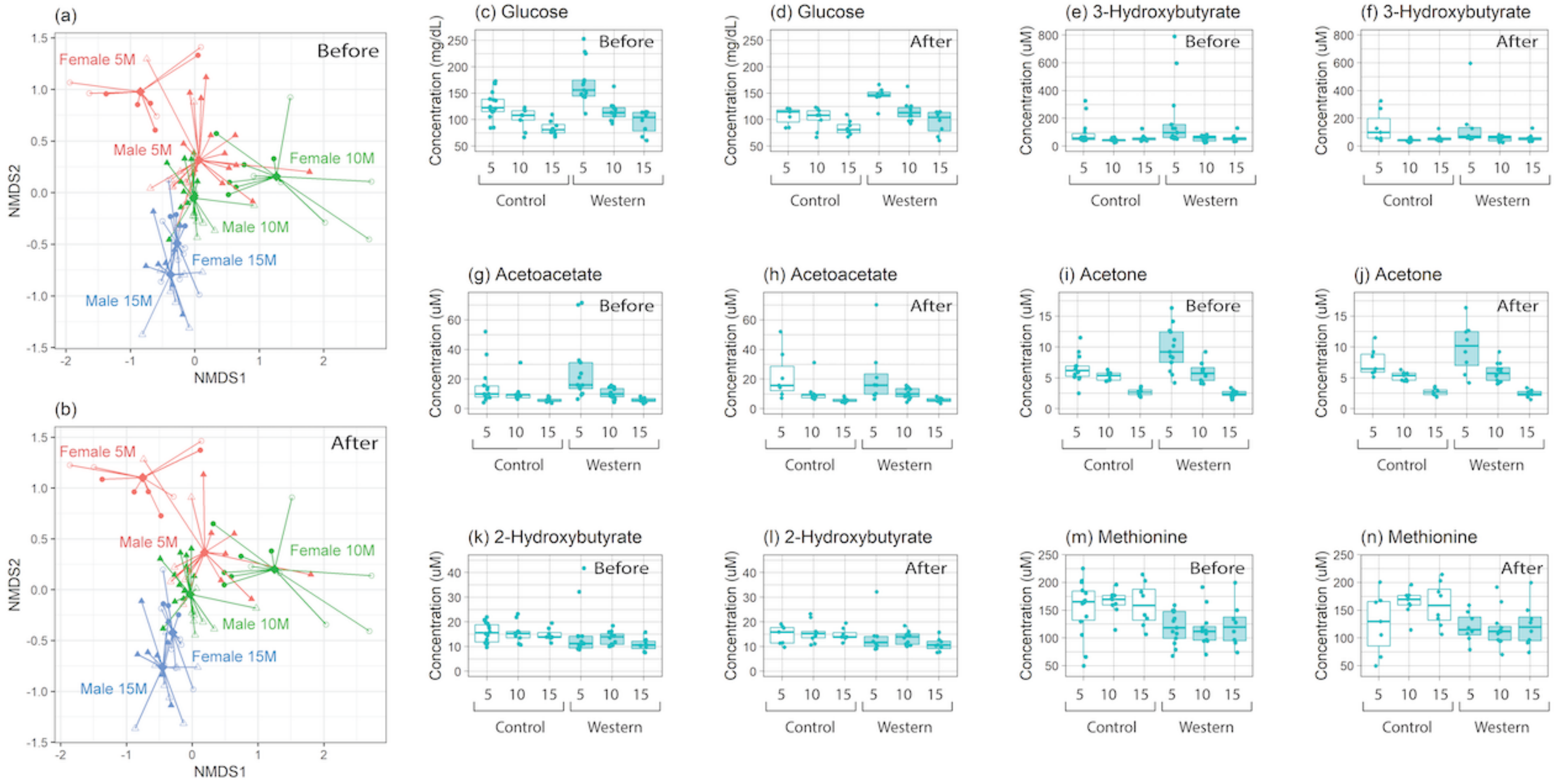

Supplementary Figure 5. NMDS plots before and after removing samples with high glucose. (a) NMDS plot generated with all samples included (“full dataset”); (b) NMDS plot generated without male samples at 5 months of age that showed high blood glucose level (“reduced dataset”). Samples collected at 5, 10, and 15 months of age are in red, green and blue respectively; samples collected from female and male are expressed with circles and triangles respectively; samples on the CD and WD are open or closed, respectively. Boxplots showing the concentrations of serum metabolites from male mice. The middle line in the box plot represents the median of data. CD samples are expressed as empty boxes and WD samples are expressed as filled boxes. Boxplots were generated using either full or reduced for (c), (d) glucose; (e), (f) 3-Hydroxybutyrate; and (g), (h) acetoacetate; (i), (j) acetone; (k), (l) 2-Hydroxybutyrate; (m), (n) methionine.

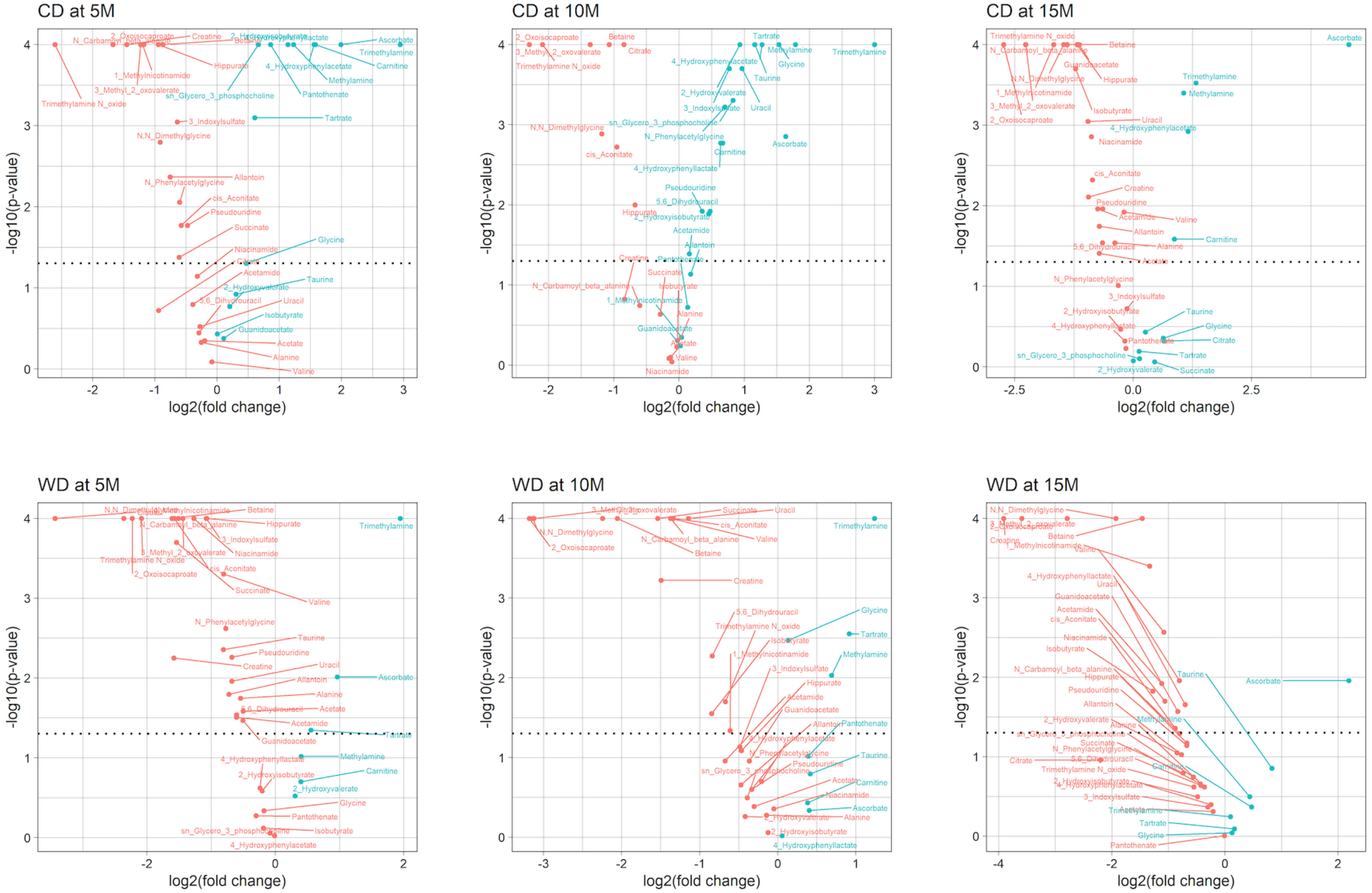

Supplementary Figure 6. Volcano plots showing the difference in the urine metabolome between male and female mice on either the CD or WD. Among 38 urine metabolites that showed significant impact of sex in Supplementary Table 3, a post-hoc test was applied to assess the impact of sex in mice on either the CD or WD at each age. Metabolites with higher levels in male mice are indicated in blue, whereas those with higher levels in female mice indicated in red. Metabolites above the dotted line correspond to those with a p-value < 0.05.

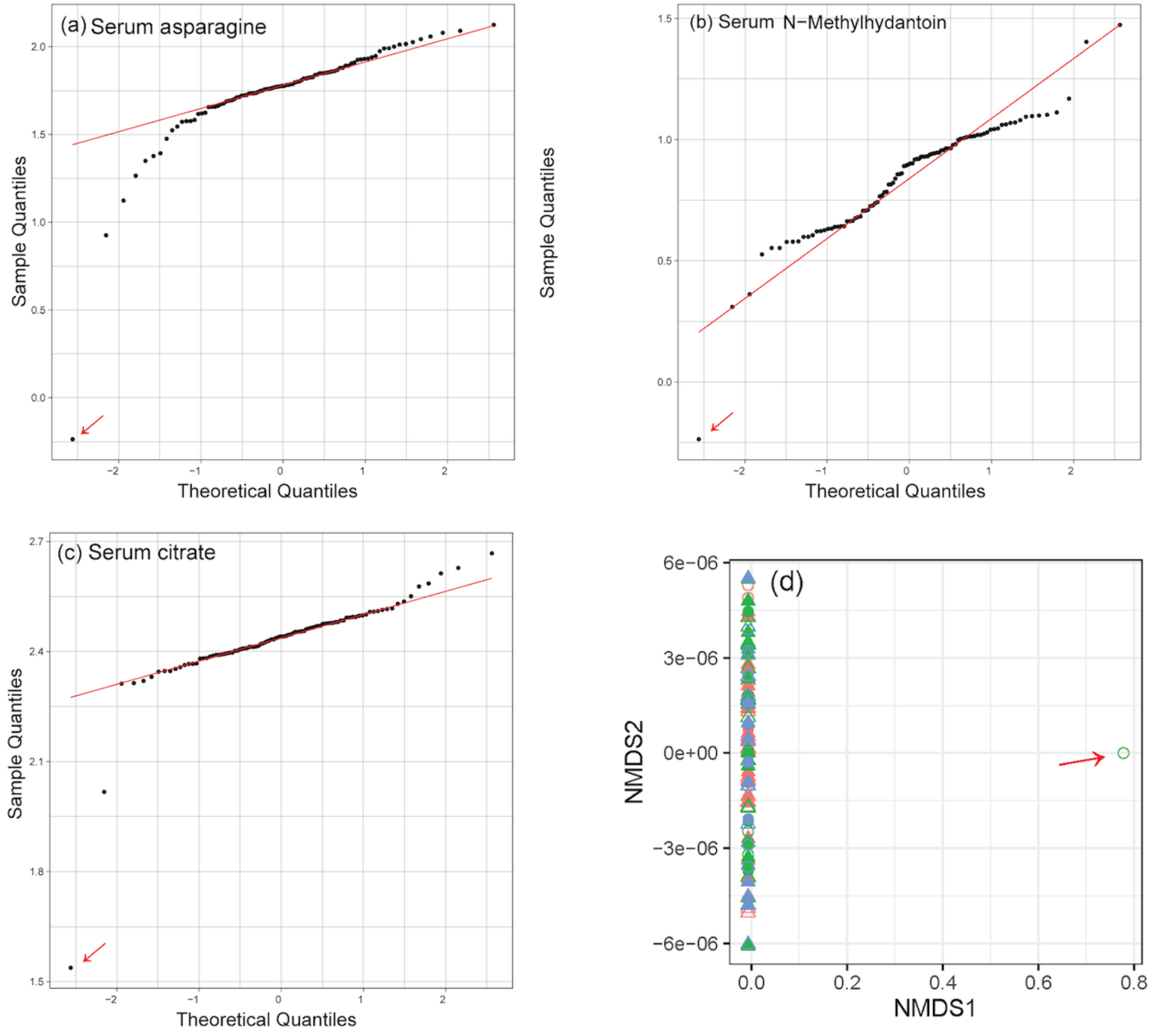

Supplementary Figure 7. Outliers removed in this study from the serum and cecal datasets (indicated with red arrows). Outliers in the serum samples were identified using QQplots: (a) asparagine; (b) N-Methylhydantoin; and (c) citrate. The outlier in the cecal microbiome dataset was identified by (d) NMDS plot.
